# Dissemination and implementation strategies for physical activity guidelines among adults with disability, chronic conditions, and pregnancy: a systematic scoping review

**DOI:** 10.1186/s12889-022-13317-3

**Published:** 2022-05-24

**Authors:** T. L. Morgan, C. Romani, A. Ross-White, A. Latimer-Cheung, J. R. Tomasone

**Affiliations:** 1 School of Kinesiology and Health Studies, Queen’s University, 28 Division Street, Kingston, ON K7L 3N6 Canada; 2 Queen’s University Bracken Health Sciences Library, Queen’s University, Kingston, ON K7L 2V5 Canada

**Keywords:** Physical activity, Systematic review/meta-analysis, Guidelines and recommendations, Health promotion, Disability, Chronic disease, Pregnancy

## Abstract

**Background:**

Physical activity guidelines for adults with disability, chronic conditions, and pregnancy (i.e., specific populations) have been developed to provide guidance for engaging in physical activity. However, specific populations remain considerably less physically active compared to the general population, presenting a knowledge-practice gap.

**Purpose:**

The purpose of this systematic scoping review was to identify and evaluate strategies for disseminating and implementing physical activity guidelines among specific populations and/or stakeholders (e.g., healthcare professionals) in Canada.

**Methods:**

Five search approaches (peer-reviewed literature databases, grey literature database, custom Google search engines, targeted web-based searches, and content expert consultation) identified records documenting and/or evaluating strategies that had been used to disseminate or implement guidelines from a predetermined list. Systematic and scoping review protocols were followed. Risk of bias assessments were conducted for all studies that evaluated strategies.

**Results:**

Eighty-one records reported dissemination strategies (*n* = 42), implementation strategies (*n* = 28), or both (*n* = 11). Twenty-two studies reporting on 29 evaluated strategies were deemed “serious” or “high” risk of bias. Common guideline dissemination and implementation strategies are deliberated and recommendations for future practice are made.

**Conclusions:**

Findings may inform future dissemination and implementation efforts for physical activity guidelines in Canada or similar countries.

**Supplementary Information:**

The online version contains supplementary material available at 10.1186/s12889-022-13317-3.

## Background

Physical activity (PA) guidelines for the general population endorse minimum levels of PA needed to achieve health benefits. However, specific populations [i.e., persons with disability and/or chronic conditions and persons who are pregnant] are typically excluded from PA guidelines for the general population as they require unique considerations to safely and effectively engage in PA [1–3]. The evidence behind general population national-level guidelines does not include population-specific evidence [4]. Accordingly, PA guidelines have been developed for 8 specific populations in Canada: spinal cord injury (SCI), cancer, multiple sclerosis (MS), osteoporosis, diabetes, Parkinson’s disease, Alzheimer’s disease, and pregnancy (Table 1). Globally, Canada is one of few countries that has established and endorsed population-specific PA guidelines, based on the preferences of Canadians from sub-populations and organizations that support population-specific (e.g., the Rick Hansen Institute) and population-wide mandates (e.g., the Canadian Society for Exercise Physiology; CSEP). Compared to the general population, PA guidelines for specific populations recommend *optimal* levels of PA needed to achieve *fitness and *health benefits. Specific populations can accrue such benefits from levels of PA that are lower than general population guideline recommendations and there is no evidence yet to suggest that a dose of 150 min of moderate-to-vigorous PA/week supports specific populations in achieving health benefits [5].

**Table 1:** Included Canadian physical activity guidelines

Author/Organization, Year	Guideline Name	Population
Cancer Care Ontario, 2015 [6]	Exercise for People with Cancer	Cancer
The American College of Sports Medicine, 2019 [7]	Exercise Guidelines for Cancer Survivors: Consensus Statement from International Multidisciplinary Roundtable	Cancer
SCI Action Canada, 2011 [8]	SCI Exercise Guidelines	SCI
SCI Action Canada, 2017^a^[9]	Scientific Exercise Guidelines for Adults with SCI	SCI
CSEP, 2013 [10]	Canadian Physical Activity Guidelines for Adults with MS	MS
CSEP, 2019 [1]	Canadian Guideline for Physical Activity Throughout Pregnancy	Pregnancy
Osteoporosis, 2014 [11]	Too Fit to Fracture	Osteoporosis
Parkinson Society Canada, 2012 [12]	Physical Activity and Parkinson’s Disease	Parkinson’s Disease
Diabetes Canada, 2018 [2]	Physical Activity and Diabetes	Diabetes
Ontario Brain Institute, 2017 [3]	Evidence-Based Messages to Promote the Use of Physical Activity to Prevent and Manage Alzheimer’s Disease	Alzheimer’s Disease

*CSEP* Canadian Society for Exercise Physiology, *MS* Multiple Sclerosis, *SCI* Spinal Cord Injury

^a^ SCI Action Canada released updated guidelines in 2017; however, given that strategies may be published for both, and 2011 falls within our record inclusion criteria (Table 2), both were included

While population-specific PA guidelines provide a basis for behaviour change, PA engagement in specific populations is low (e.g., 12% of adults with SCI meet PA guideline recommendations) [13]. High rates of inactivity among specific populations in Canada have been rising over recent years [14] and have been reported as consistently higher than the general population [15–20]. Given physical inactivity can substantially impact one’s health status [21], it is unsurprising to see high rates of poor health outcomes reported among specific populations [15, 16].

To bridge this knowledge-to-practice gap, guidelines must be accompanied by appropriate dissemination (i.e., purposive distribution of a guideline to a specific audience to enhance awareness, attitudes, and knowledge of a guideline [22]) and implementation (i.e., actions to support individuals in meeting PA guideline benchmarks to enhance self-efficacy, intention, and behaviour in line with a guideline [22]) strategies. Current knowledge of dissemination and implementation (D&I) strategies for PA guidelines is low. Tomasone et al.’s [23] recent systematic scoping review examined D&I strategies of movement guidelines (i.e., PA, sleep, and/or sedentary behaviour) for the general population in Canada and similar high-income countries. Despite the inclusion of 15 guidelines and an extensive search, only 47 records were included [23]. Dissemination strategies were more common than implementation strategies, yet implementation strategies were more likely to be evaluated and show positive changes in guideline-specified behaviour [23]. However, population-specific guidelines were excluded from this review.

Compared to the general public, specific populations represent a smaller subset of the Canadian population [24, 25] and have specialized health professionals who could uniquely target them (e.g., oncologists, midwives). Thus, strategies for disseminating and/or implementing PA guidelines among specific populations may be more tailored and more likely to be effective [26]. Population-specific PA guideline development efforts are also led by research groups and/or organizations who have established networks with specific populations [6, 27, 28], which may facilitate ease of guideline D&I relative to general population efforts. Moreover, PA guidelines for specific populations are often funded by special interest group research grants, which mandate end-of-grant dissemination and/or implementation endeavours. Thus, we expect greater PA guideline D&I initiatives among specific populations compared to the general population. As the evidence base for key guideline D&I strategies is emergent, population-specific initiatives could provide models for enhancing D&I efforts of general population guidelines, which may also be transferrable to international multi-population guidelines, such as the World Health Organization 2020 guidelines on PA and sedentary behaviour [29]. Accordingly, a systematic scoping review to examine D&I strategies for PA guidelines among specific populations was deemed necessary.

The purpose of this systematic scoping review was to identify and evaluate strategies used for the D&I of PA guidelines among adults 18 + years of specific populations and/or stakeholders (e.g., healthcare professionals) in Canada. Informed by Tomasone and colleagues’ [23] review, research questions (RQs) were formulated for both the D&I of included guidelines.

For guideline dissemination: (1) what strategies have been used in Canada to disseminate PA guidelines for specific populations? (2) of the dissemination strategies identified, how have they been evaluated? and (3) of the dissemination strategies evaluated, which were effective in improving guideline awareness, attitudes and knowledge?

For guideline implementation: (4) what strategies have been used in Canada to implement PA guidelines for specific populations? (5) of the implementation strategies identified, how have they been evaluated? and (6) of the implementation strategies evaluated, which were effective in improving self-efficacy, intention and behaviour in line with the recommendation, and self-efficacy and intent to use the guideline?

## Methods

Guideline D&I reports are commonly published in locations other than peer-reviewed journals [23]; thus, a systematic scoping review was deemed the most appropriate study design for this investigation. Systematic scoping reviews amalgamate the rigorous and exploratory methods of systematic reviews and scoping reviews, respectively [30]. A search strategy was modelled after Tomasone et al. [23] and recommendations by D’Urzo et al. [30] for systematic scoping review conduct, and the Joanna Briggs Institute [31] and other established scoping review frameworks [32] to ensure that our approach to identifying non-peer reviewed records was comprehensive. The protocol for this review was registered in Open Science Framework on 13 February 2020 (https://osf.io/2h875). The present review is reported in line with the Preferred Reporting Items for Systematic reviews and Meta-Analyses extension for Scoping Reviews (PRISMA-ScR) [33].

### Eligibility criteria

Guidelines and/or recommendations (i.e., statements informed by a systematic review of evidence or based on expert appraisal of synthesized evidence, respectively) [34] for improving fitness levels or health status through PA and/or exercise among specific populations were identified. Eligible guidelines were those released in the past 10 years in Canada or those intended for use in Canada. Guidelines international in scope must have been led by a researcher working in Canada and endorsed by a Canadian organization that promotes PA for eligibility in this study. Guidelines were restricted to the English language due to a lack of funding for translation. Guidelines were identified through targeted website searching followed by a survey of published literature (Supplement 1) and were reviewed by all authors. Note that authors four and five have established research programs that include PA guideline development and promotion efforts for several of the included populations. All 10 guidelines were deemed relevant (Table 1).

### Record identification

Records were sought via published literature, organizational releases, guideline messaging, web-pages, and any other records meeting pre-defined eligibility criteria (Table 2). Records must have reported a strategy for disseminating and/or implementing one of the PA guidelines in Table 1 and targeted adults (18 + years) of the identified specific population or their stakeholders (i.e., healthcare professionals). Included records must have used a human participant pool, been delivered in English, and been published from 2011 onwards. This date reflects recent advancements in the field of dissemination, whereby a greater range of strategies (e.g., social media) are being used for health promotion [35]. All study designs were considered in answering RQs 1, 2, 4, and 5. Experimental, quasi-experimental, pre/post, and prospective designs were considered in answering RQs 3 and 6.

**Table 2:** Record inclusion and exclusion criteria

Inclusion	Exclusion
• Reports one or more strategy for the dissemination or implementation of a guideline listed in Table 1	• Reports strategies for the dissemination or implementation of a guideline not listed in Table 1
• Record published in 2011 or later	• Record published before 2011
• Strategy is delivered in Canada	• Strategy is delivered outside Canada
• Record written in English	• Record written in a language other than English
• Strategy targets adults 18 + years old belonging to a specific population^a^, or stakeholders of the specific population (e.g. a healthcare professional)	• Strategy targets individuals under the age of 18 belonging to a specific population or stakeholders

^a^Specific populations were defined as any population requiring unique considerations to safely and effectively engaging in PA compared to the general population, who were excluded from general population guidelines [i.e., persons who are pregnant; individuals with disease-specific conditions (e.g., diabetes, cancer), a functional impairment limiting mobility, or a need for hospital-based or long-term care] [4].

### Search strategy

In line with Tomasone et al. [23] and D’Urzo et al., [30] five search approaches were used: (1) peer-reviewed literature databases [Web of Science (Web of Science Core Collection, BIOSIS Previews, KCI-Korean Journal Database, MEDLINE®, Russian Science Citation Index, SciELO Citation Index) and Google Scholar (Publish or Perish (https://harzing.com/resources/publish-or-perish)] [36], (2) grey literature database (Thesis & Dissertation – ProQuest Dissertations Online), (3) custom Google search engines [Carleton University’s MADGIC search engine (http://subject-guides.uwaterloo.ca/c.php?g=695548&p=4931873) and Ontario Public Health Libraries Association (http://www.ophla.ca/resources.htm)], (4) targeted web-based searches, and (5) content expert consultation. Peer-reviewed literature searches, grey literature searches, and custom Google searches were developed and carried out by a professional librarian (third author). The peer-reviewed literature searches were conducted in Web of Science databases as the cited reference feature was used to find articles that referenced the pre-specified list of guidelines (Table 1). Unlike traditional reviews, which find limited value in citation searching, this method was preferred as it is unlikely that articles about guideline D&I fail to reference the guideline in question. Targeted web-based searches were carried out by the second author and audited by the first author. Search terms (Supplement 1) were modified per data source but remained specific to the pre-specified guidelines (Table 1) and to dissemination and/or implementation. The first four search approaches took place from November 2019 to March 2020.

For the targeted web-based searches, the website for the publishing organization of each guideline in Table 1, and for organizations that promote PA to specific populations in Canada (e.g., ParticipACTION), were searched. Additional websites were identified by entering each guideline name into the search bar and scanning the first 100 hits. Two methods were used to identify potential reports of guideline D&I strategies: (i) the 2-click method (i.e., records within 2 clicks of the guideline page were included), and (ii) search strings were developed and run through website search bars (Supplement 1).

The content expert consultation was conducted following the completion of other searches to identify any additional records for each guideline that were potentially missed or not publicly available. Primary authors involved in the development of each guideline were identified as potential content experts. Where no primary author could be identified, the publishing organization was contacted directly (i.e., Diabetes Canada, Parkinson Canada). Where a single researcher led multiple guidelines, one email was sent regarding all relevant guidelines. Initial emails were sent to 8 content experts in February 2020 and included a copy of Table 1 and any reports of dissemination and/or implementation of the guideline identified through the four other search methods. Experts were asked to provide (a) PDFs, citations, and/or links to published manuscripts, or (b) (un)published organizational reports of guideline dissemination and/or implementation, and to nominate an alternate contact if they felt that individual could better respond to the request. Two weeks from initial contact, a reminder was sent offering an additional two weeks to respond. In total, 11 content experts (representing all 10 guidelines) were contacted, from which 10 responses (representing all but the pregnancy guidelines) were received.

### Record selection

Published literature, grey literature, and custom Google search results were screened using the Covidence software [37]. The first and second author independently screened each title and abstract for potential eligibility. Specific to targeted web-based searches, titles of up to the first 100 records from each search were screened [30]. Relevant titles and their corresponding URLs were compiled in a Microsoft Excel spreadsheet for full-text screening.

Full-text screening for peer reviewed literature, grey literature, and custom Google search results was performed by the first and second authors. Discrepancies were resolved at both stages through consultation between the two reviewers and the last author where necessary. Full-texts identified by targeted web-based searches and content experts were screened by the second author and audited by the first author.

### Collating, summarizing and reporting the results

The data extraction table in the present review was modelled after Tomasone et al. [23] to extract data pertaining to record characteristics (i.e., title, year, author, participant characteristics, and study design), intervention characteristics (i.e., setting, relevant guideline, strategy and evaluation format if evaluated), dissemination outcomes (i.e., awareness, attitude, and knowledge of the guideline and any other non-specified dissemination outcome), and implementation outcomes (i.e., self-efficacy, intentions, and/or behaviours relating to the guideline, and any other non-specified implementation outcome). The data extraction table was pilot tested by the second author and audited by the first author.

Extracted strategies were placed into seven classifications according to Tomasone et al.’s [23] framework for movement guideline D&I strategies (Table 3), which was adapted from Leeman et al.’s [38] framework for classifying implementation strategies for evidence-based healthcare interventions.

**Table 3:** Physical activity guideline dissemination and implementation strategy classifications (modified from Tomasone et al., 2020b)

Classification	Definition	Actor Examples	Outcomes	Strategy Type (*Definition)*	Examples from the current review
Dissemination process strategies	Enacted by those working within delivery systems and pertain to processes or activities that dissemination teams perform to plan, select, and disseminate a guideline	Delivery system^a^ • Researchers • Policymakers • Guideline development organizations	Extent, quality and timeliness of the completion of activities related to specific dissemination process strategies	Formative research (*the process of developing effective strategies and communication channels, and understanding the attributes of the target population to enhance guideline reach and uptake*) [39]	*n* = 6 [40–45]
Dissemination strategies	Any action(s) that distributes the guideline to actors in the delivery system, support system, and synthesis and translation system’s awareness, knowledge, attitudes, and intention to adopt a guideline	Delivery system^a^ • Members of the general public • Health professionals Support system^b^ • Researchers • Policy makers Synthesis and translation systems^c^ • Guideline development organizations • Funding organizations	Awareness, knowledge, attitudes, and intention to adopt a guideline	Distribution of guideline materials (*scientific or public-facing documents disseminated electronically, audio-visually, or by hard-copy*) [46]. Example: fact sheet (i.e., one or more pages including factual information required to perform or understand a target behaviour) [47]	*n* = 30 [8, 12, 48–75]
				Mass media/communications campaign (*widespread, intentional distribution of information to a population using pre-existing media venues, such as internet, television, or print media, which may be embedded within a broader marketing approach*) [76]	*n* = 9 [50, 51, 77–83]
				Education (*teaching about the benefits of complying with a guideline from a credible and capable source*) [84]	*n* = 10 [85–94]
Dissemination scale-up strategies	Enacted by support system actors with the goal of getting multiple settings to disseminate a guideline	Support system^b^ • Researchers • Policy makers • Guideline development organizations	Motivation and capacity to disseminate, and actual dissemination of, a guideline across multiple settings	Dissemination toolkits (*a set of resources that can be used individually or collectively to enhance communication and dissemination in more than one setting*) [95]	*n* = 0
Implementation process strategies	Enacted by those working within delivery systems and pertain to processes or activities that implementation teams perform to plan, select, and integrate a guideline into practice	Delivery system^a^ • Researchers • Policy makers	Extent, quality and timeliness of the completion of activities related to specific implementation process strategies	Engaging stakeholders (*actively petitioning involvement from individuals or groups who have a vested interest in a research/policy project to enhance collaboration and decision-making*) [96]	*n* = 5 [43, 52, 97–99]
				Human resources (*increasing the number, or qualifications, of staff to facilitate guideline implementation*) [46]	*n* = 4 [42, 100–102]
Integration strategies	Delivered by actors within delivery systems and include any action(s) that influence the integration of a specific guideline into practice	Delivery system^a^ • Members of the general public • Health professionals • Researchers • Not for profit organizations	Self-efficacy and intention to use the guideline, and self-efficacy, intention and behaviour in line with the guideline recommendations	Feedback (*data reported to an individual on their current level of adherence to behavioural guideline benchmarks in aims of improving their compliance*) [46]	*n* = 1 [103]
				Alerts (*providing audio-visual prompts to remind individuals to comply with, or to signal deviation from, a guideline benchmark*) [46]	*n* = 1 [103]
				Financial incentives (*direct or indirect financial reward for complying with behaviour that meets guideline benchmarks*) [46]	*n* = 1 [103]
				Skills training (*the provision of various techniques, including instructions on how to perform the behaviour, behavioural rehearsal, and demonstration of the behaviour, to enable an individual to meet behavioural guideline benchmarks*) [104]	*n* = 5 [42, 98, 100–102]
				Counselling (*an approach that incorporates various strategies to assess an individual’s current behaviour, provide education, goal-setting, and resources, and monitor behavioural improvements*) [105]	*n* = 7 [42, 98, 106–110]
				Planning tools (*instruments that can be used to guide actionable steps that help individuals meet guideline benchmarks*) [46]	*n* = 16 [12, 42, 49, 54, 69, 75, 86, 87, 93, 103, 106, 111–115]
Capacity building strategies	Delivered by support systems and target individuals’ general capacity (motivation, self-efficacy) to execute implementation process strategies	Support system^b^ • Researchers • Policy makers • Guideline development organizations	Self-efficacy and motivation to engage in implementation process strategies, and completion of implementation process strategies	Stakeholder training (*one or more workshops/courses/seminars to increase stakeholders’ knowledge, ability, and operational capacity to deliver a given process strategy*) [116]	*n* = 6 [97, 107, 117–120]
Scale-up strategies	Enacted by support system actors with the goal of getting multiple settings to implement a guideline. Scale up strategies target determinants at multiple levels (i.e., from end user to policy)	Support system^b^ • Researchers • Policy makers • Guideline development organizations	Motivation and capacity to implement, and actual implementation of, a guideline across multiple settings	Implementation toolkits (*a set of tools that can be used individually or collectively to enhance implementation in more than one setting*) [121]	*n* = 7 [92, 122–127]

^a^Delivery systems include the individuals, teams, and systems that adopt and integrate a guideline into practice

^b^Support systems include individuals, teams, and systems that build a delivery system's capacity to adopt and integrate a guideline into practice

^c^Synthesis and translation systems include the organizations that identify, translate, and disseminate guidelines

Following data extraction, records were synthesized into two tables, representing D&I strategies. Within each table, records were organized by strategy class (e.g., dissemination process strategy, implementation scale-up strategy), then by strategy type (e.g., distribution of guideline materials, mass media/communication campaigns), then by number of RQs addressed, then by the guideline they aimed to disseminate or implement.

As anticipated, heterogeneity of results pertaining to the effectiveness of strategies precluded the utilization of meta-analysis.

### Risk of Bias

A risk of bias (ROB) assessment was conducted for all records that evaluated the effectiveness of a dissemination or implementation strategy (i.e., RQs 3 and/or 6). ROB assessments were conducted by the second author and audited by the first author. Randomized controlled trial (RCT) study designs were assessed according to the Cochrane Collaboration ROB tool [128]. Non-randomized study designs were assessed according to the Cochrane ROB Assessment Tool for Non-Randomized Studies [129].

## Results

### Record characteristics

**Fig. 1:**
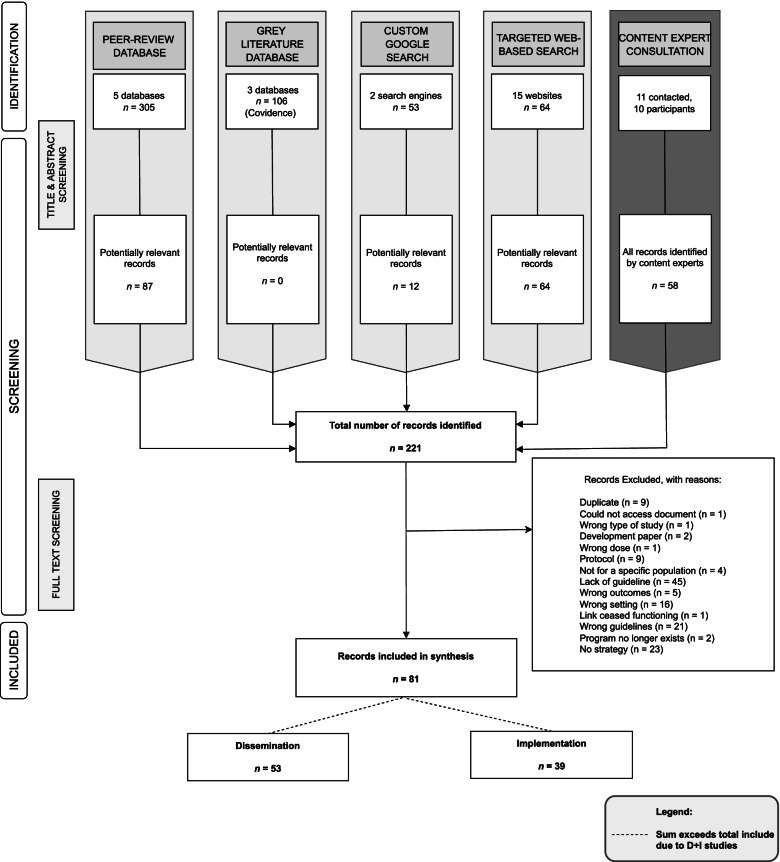
PRISMA diagram of study flow^*a*^

Peer reviewed literature searches, grey literature searches, custom Google search engines and targeted-web search approaches identified 528 records. As Web of Science’s cited reference feature allowed us to search multiple databases simultaneously, duplicates from these databases were not recorded. With grey literature searches, duplicates were also not recorded. Thus, zero duplicates were recorded during title and abstract screening. Of the 528 records, 163 records were deemed potentially relevant and were retrieved for full-text screening. Of the 11 contacted content experts, 10 participated and contributed an additional 58 records, all of which were retrieved for full-text screening. The full texts of 221 records were screened and 81 were included in data synthesis (Fig. 1).

The content expert consultation search approach had the highest yield at 44 unique records, followed by targeted web-based searches (*n* = 27). All other records were identified through peer-reviewed and grey literature searches (*n* = 10). The custom Google searches identified zero records.

Of the 81 records, 79 pertained to single guidelines while two related to two guidelines (i.e., for cancer survivors and pregnancy) [77, 78]. Forty-two records reported only dissemination strategies, 28 reported only implementation strategies, and 11 reported both D&I strategies, for a total of 53 records discussing dissemination strategies and 39 discussed implementation strategies. There were 23 instances where multiple strategies were used in a single record (e.g., “counselling” and “planning tools”) [106], resulting in 109 instances of the use of a strategy. Members of specific populations were targeted in 51 records, stakeholders (e.g., healthcare professionals) were targeted in 23 records, and 7 records targeted both.

### Dissemination

#### RQ1: What strategies have been used in Canada to disseminate PA guidelines for specific populations?

Of the 53 dissemination records, six records identified six dissemination process strategies. The remaining 47 records identified a total of 49 dissemination strategies, with two records having used more than one strategy. No records identified dissemination scale-up strategies. Thus, a total of 55 instances of dissemination strategies were identified. Of the 6 dissemination process strategies, all were categorized as “formative research”. For example, the Ontario Brain Institute conducted surveys regarding end-user perceptions of the benefits, appropriateness, and overall usefulness of the Alzheimer’s recommendations [40].

Of the 49 dissemination strategies identified, the most common strategy was “distribution of guideline materials” (*n *= 30). For example, CSEP released their scientific statement on the PA guidelines for MS on their website [48]. Next was “education” (*n *= 10), such as Diabetes Canada’s information pamphlets on the importance of engaging in resistance and aerobic exercise and how to progress through resistance training [49, 85–88]. The least common dissemination strategy was “mass media/communications campaigns” (*n* = 9). For instance, a New York Times article explained the benefits of exercise for cancer survivors, including advice from experts in the field.

Two records used two dissemination strategies [50, 51]. For example, a podcast functioned both to distribute guideline materials and as a mass media/communications campaign to disseminate the guidelines for cancer survivors [50].

#### RQ2: Of the dissemination strategies used, how have they been evaluated?

Of the 55 dissemination strategies, six (11%) included an evaluation (2 dissemination process strategies [40, 41]; 4 dissemination strategies) [52, 89–91]. Surveys were the most common evaluation method (*n *= 5), but two studies used semi-structured interviews [52, 91]. Dissemination process outcomes included the quality of completion of activities related to the process strategy [40, 41]. Dissemination outcomes included guideline awareness [52, 90], knowledge [90], and attitudes [89]. Outcomes beyond those specified in RQ3 are reported in Table 4.

**Table 4:** Dissemination strategies identified

Record	Guideline	RQ1: Strategy		RQ2: Evaluation	RQ3: Outcomes	ROB
		**Type**	**Description**			
**DISSEMINATION PROCESS STRATEGIES (*n* = 6)**						
Antflick (2014) [40]	Formulation of evidence-based messages to promote the use of physical activity to prevent and manage Alzheimer’s disease (2017) [3]	Formative research	End-users provided feedback on the usefulness of the recommendation statement	Cross-sectional survey	Participants felt the toolkit provided appropriate information to help older adults become more active (4.21/5), useful info for people with Alzheimer’s disease or who want to prevent Alzheimer’s disease (4.14/5), clear info on the benefits of PA for preventing (4/5) and managing (3.93/5) Alzheimer’s disease	Serious^c^
Antflick (n.d.) [41]	Formulation of evidence-based messages to promote the use of physical activity to prevent and manage Alzheimer’s disease (2017) [3]	Formative research	Physicians provided feedback on the usefulness of the recommendation statement	Cross-sectional survey	Participants felt neutral regarding confidence that a client with Alzheimer’s disease could engage in enough PA to meet current guidelines (3/5); given the opportunity they would use the messaging statement to recommend PA (4/5); majority felt the toolkit provides useful information for HCP (4.8/5) and is appropriate for all community dwelling adults with Alzheimer’s disease (4.4/5)	Serious^c^
Latimer-Cheung (2013) [42]	Canadian Physical Activity Guidelines for Adults with MS (2013) [10]	Formative research	Agenda for meeting with objectives to produce recommendations for format and content of toolkit to supplement PA guidelines for people with MS, and propose toolkit dissemination strategies	N/A	N/A	N/A^d^
Latimer-Cheung & Martin Ginis (n.d.) [44]	Canadian Physical Activity Guidelines for Adults with MS (2013) [10]	Formative research	Describes the development process of e-modules to enhance dissemination and uptake of the MS guidelines	N/A	N/A	N/A^d^
Shirazipour (2013) [43]	Canadian Physical Activity Guidelines for Adults with MS (2013) [10]	Formative research	Meeting minutes from consensus panel to develop and disseminate an evidence-based toolkit to inform adults of the guidelines and teach them to make smart, informed choices about PA and goal setting (topics included format and use of photos, etc.)	N/A	N/A	N/A^d^
Latimer-Cheung (2013) [45]	Canadian Physical Activity Guidelines for Adults with MS (2013) [10]	Formative research	Engaging and informing stakeholders throughout the development and dissemination of the MS Get Fit Toolkit	N/A	N/A	N/A^d^
**DISSEMINATION STRATEGIES (*n* = 49)**						
Clark et al. (2017) [52]	Too Fit to Fracture (2014) [11]	Distribution of guideline materials	Too Fit to Fracture recommendation summary was mailed to each physician/NP prior to interviews	Cross-sectional focus groups and semi-structured interviews	23.7% of participants had prior awareness of the Too Fit to Fracture recommendations	Serious^c^
Osteoporosis Canada (n.d.-b) [75]	Too Fit to Fracture (2014) [11]	Distribution of guideline materials	Public facing statement (fact sheet, tips, guides, resources)	N/A	N/A	N/A^d^
Diabetes Canada (n.d.-a) [49]	Physical Activity and Diabetes (2018) [2]	Distribution of guideline materials	Public facing statement (Fact sheet)	N/A	N/A	N/A^d^
Diabetes Canada (n.d.-g) [55]	Physical Activity and Diabetes (2018) [2]	Distribution of guideline materials	Public facing statement (interactive tools and resources)	N/A	N/A	N/A^d^
^a^Campbell (2019) [51]	Exercise Guidelines for Cancer Survivors: Consensus Statement from International Multidisciplinary Roundtable (2019) [7]	Distribution of guideline materials	A series of 9 Twitter posts sharing the scientific report for professionals	N/A	N/A	N/A^d^
^a^ Campbell & Winter-Stone (2019) [50]	Exercise Guidelines for Cancer Survivors: Consensus Statement from International Multidisciplinary Roundtable (2019) [7]	Distribution of guideline materials	Podcast discussing and providing a link to the scientific report for professionals	N/A	N/A	N/A^d^
American College of Sports Medicine (2019a) [56]	Exercise Guidelines for Cancer Survivors: Consensus Statement from International Multidisciplinary Roundtable (2019) [7]	Distribution of guideline materials	Public facing statement (fact sheet, poster)	N/A	N/A	N/A^d^
American College of Sports Medicine (2018) [57]	Exercise Guidelines for Cancer Survivors: Consensus Statement from International Multidisciplinary Roundtable (2019) [7]	Distribution of guideline materials	Public facing statement (infographic, fact sheet)	N/A	N/A	N/A^d^
Exercise is Medicine (n.d.-a) [73]	Exercise Guidelines for Cancer Survivors: Consensus Statement from International Multidisciplinary Roundtable (2019) [7]	Distribution of guideline materials	Public facing statement (infographic)	N/A	N/A	N/A^d^
Sunnybrook Odette Cancer Centre (2016) [58]	Exercise for People with Cancer (2015) [6]	Distribution of guideline materials	Public facing statement (fact sheet with guideline information and frequently asked questions and answers)	N/A	N/A	N/A^d^
Trillium Health Partners (n.d.) [59]	Exercise for People with Cancer (2015) [6]	Distribution of guideline materials	Public facing statement (fact sheet)	N/A	N/A	N/A^d^
Cancer Care Ontario (n.d.-a) [61]	Exercise for People with Cancer (2015) [6]	Distribution of guideline materials	Public facing statement (posters)	N/A	N/A	N/A^d^
Cancer Care Ontario (n.d.-b) [60]	Exercise for People with Cancer (2015) [6]	Distribution of guideline materials	Public facing statement (fact sheet)	N/A	N/A	N/A^d^
Cancer Care Ontario (n.d.-c) [62]	Exercise for People with Cancer (2015) [6]	Distribution of guideline materials	Public facing statement (fact sheet)	N/A	N/A	N/A^d^
SCI Action Canada (2011a) [8]	SCI Exercise Guidelines (2011) [8]	Distribution of guideline materials	Public facing statement	N/A	N/A	N/A^d^
SCI Action Canada (2011b) [53]	SCI Exercise Guidelines (2011) [8]	Distribution of guideline materials	Public facing statement (key messages about the guidelines)	N/A	N/A	N/A^d^
SCI Action Canada (2011c) [54]	SCI Exercise Guidelines (2011) [8]	Distribution of guideline materials	Public facing statement (fact sheet, print pamphlet)	N/A	N/A	N/A^d^
SCI Action Canada (2018) [74]	SCI Exercise Guidelines (2011) [8]	Distribution of guideline materials	Scientific statement	N/A	N/A	N/A^d^
SCI Action Canada (2019a) [66]	Scientific Exercise Guidelines for Adults with SCI (2017) [9]	Distribution of guideline materials	Public facing statement (fact sheet, frequently asked questions and answers)	N/A	N/A	N/A^d^
SCI Action Canada (2019b) [67]	Scientific Exercise Guidelines for Adults with SCI (2017) [9]	Distribution of guideline materials	Public facing statement	N/A	N/A	N/A^d^
Canadian Society for Exercise Physiology (n.d.-a) [68]	Canadian Physical Activity Guidelines for Adults with MS (2013) [10]	Distribution of guideline materials	Public facing statement (fact sheet, frequently asked questions and answers)	N/A	N/A	N/A^d^
Canadian Society for Exercise Physiology (n.d.-b) [48]	Canadian Physical Activity Guidelines for Adults with MS (2013) [10]	Distribution of guideline materials	Scientific statement	N/A	N/A	N/A^d^
MS Society of Canada (n.d.-b) [69]	Canadian Physical Activity Guidelines for Adults with MS (2013) [10]	Distribution of guideline materials	Public facing statement presented within the MS Get Fit Toolkit	N/A	N/A	N/A^d^
Maclaren (2018) [65]	Canadian Guideline for Physical Activity Throughout Pregnancy (2019) [1]	Distribution of guideline materials	Public facing statement	N/A	N/A	N/A^d^
Ontario Brain Institute (2014a) [63]	Formulation of evidence-based messages to promote the use of physical activity to prevent and manage Alzheimer’s disease (2017) [3]	Distribution of guideline materials	Public facing statement (fact sheet)	N/A	N/A	N/A^d^
Grimes et al. (2019) [64]	Physical Activity and Parkinson’s Disease (2012) [12]	Distribution of guideline materials	Scientific statement and public facing statement (poster, fact sheet)	N/A	N/A	N/A^d^
Parkinson Canada (2018) [71]	Physical Activity and Parkinson’s Disease (2012) [12]	Distribution of guideline materials	Public facing statement	N/A	N/A	N/A^d^
Parkinson Canada (2015) [70]	Physical Activity and Parkinson’s Disease (2012) [12]	Distribution of guideline materials	Public facing statement (fact sheet)	N/A	N/A	N/A^d^
Parkinson Society Canada (2012) [12]	Physical Activity and Parkinson’s Disease (2012) [12]	Distribution of guideline materials	Public facing statement (fact sheet)	N/A	N/A	N/A^d^
Parkinson Canada (n.d.-a) [72]	Physical Activity and Parkinson’s Disease (2012) [12]	Distribution of guideline materials	Public facing statement (educational resources, fact sheets)	N/A	N/A	N/A^d^
Hutchinson (2020) [81]	Exercise Guidelines for Cancer Survivors: Consensus Statement from International Multidisciplinary Roundtable (2019) [7]	Mass media/communications campaign	Online article in The Globe and Mail	N/A	N/A	N/A^d^
^a^Campbell (2019) [51]	Exercise Guidelines for Cancer Survivors: Consensus Statement from International Multidisciplinary Roundtable (2019) [7]	Mass media/communications campaign	A series of 9 Twitter posts sharing the scientific report for professionals (discusses evidence review etc.)	N/A	N/A	N/A^d^
^a^Campbell & Winter-Stone (2019) [50]	Exercise Guidelines for Cancer Survivors: Consensus Statement from International Multidisciplinary Roundtable (2019) [7]	Mass media/communications campaign	Podcast discussing the scientific report for professionals	N/A	N/A	N/A^d^
Reynolds (2019) [82]	Exercise Guidelines for Cancer Survivors: Consensus Statement from International Multidisciplinary Roundtable (2019) [7]	Mass media/communications campaign	Online article in The New York Times	N/A	N/A	N/A^d^
Devlin (2019) [80]	Exercise Guidelines for Cancer Survivors: Consensus Statement from International Multidisciplinary Roundtable (2019) [7]	Mass media/communications campaign	CTV news article	N/A	N/A	N/A^d^
Canadian Society for Exercise Physiology [@csep_scpe] (2019)[78]	Exercise Guidelines for Cancer Survivors: Consensus Statement from International Multidisciplinary Roundtable (2019) [7] + Canadian Guideline for Physical Activity Throughout Pregnancy (2019) [1]	Mass media/communications campaign	Social media (Instagram) posts about new guideline release	N/A	N/A	N/A^d^
Canadian Society for Exercise Physiology [@CSEPdotCA] (2019) [77]	Exercise Guidelines for Cancer Survivors: Consensus Statement from International Multidisciplinary Roundtable (2019) [7] + Canadian Guideline for Physical Activity Throughout Pregnancy (2019) [1]	Mass media/communications campaign	Social media (Twitter) posts about new guideline release	N/A	N/A	N/A^d^
Segal (2017) [83]	Exercise for People with Cancer (2015) [6]	Mass media/communications campaign	Powerpoint presentation for professionals about the guidelines	N/A	N/A	N/A^d^
Cancer Care Ontario (2015) [79]	Exercise for People with Cancer (2015) [6]	Mass media/communications campaign	Event catering to multidisciplinary stakeholders to better understand the recommendations and provide input	N/A	N/A	N/A^d^
Shirazipour et al. (2019) [90]	SCI Exercise Guidelines (2011) [8]	Education	Participants were surveyed on the impact of CMCL sessions on their knowledge of the guideline and the perceived utility of event-based interventions to enhance guideline knowledge	One arm, pre-post intervention using surveys	Initial guideline awareness was 17.8% for HCP and 8.9% for trainees. Of those who were aware, initially 4.3% of HCP and 15.1% of trainees could accurately recall the guideline NS differences in HCP awareness or recall from pre-to-post or from pre-intervention to 6-month follow-up. Significant decrease in HCP awareness from post-intervention to 1-month follow-up (*p* < 0.001) Significant increase in trainee awareness from pre-to-post and post intervention to 1-month follow up, but significant decrease in trainee awareness from post intervention to 6-month follow up (*p* < 0.001). Trainee recall significantly improved from pre- to post-intervention (*p* < 0.001)	Serious^b^
Smith et al. (2015) [91]	SCI Exercise Guidelines (2011) [8]	Education	Playing an evidence-based story about the process of becoming active following SCI	Cross sectional; inductive thematic analysis conducted on semi-structured telephone interviews	Participant groups felt the narrative tool was highly effective in communicating synthesized information to the right people, getting info across and providing good info about PA. They all felt the stories were authentic, relevant, accurate, truthful and credible	Serious^c^
Lithopoulos et al. (2017) [89]	Canadian Physical Activity Guidelines for Adults with MS (2013) [10]	Education	Examination of the effects of risk information, and gain-framed or loss-framed messages on perceptions of the messages and levels of PA	RCT using surveys (message perceptions, attitudes about the guidelines, intention to meet guidelines, LTPAQ-SCI, Adapted Exercise Self-efficacy Scale)	NS difference in message perceptions or intention to meet PA guidelines over the next 2 weeks. Participants overall engaged in more minutes/week of PA (+ 8.91; *p* = 0.01) and reported greater response efficacy to meet the guidelines (*p* = .001) post-intervention compared to pre-intervention. Those who received risk information reported more PA than those who did not (*p* = 0.02). NS difference was seen in task efficacy to meet the guidelines	High^b^
Ma et al. (2018) [92]	SCI Exercise Guidelines (2011) [8]	Education	Document to teach HCPs about promoting the guidelines	N/A	N/A	N/A^d^
SCI Action Canada (n.d.-a) [93]	SCI Exercise Guidelines (2011) [8]	Education	Education for end-users on how to meet guideline recommendations	N/A	N/A	N/A^d^
Osteoporosis Canada (n.d.-a) [94]	Too Fit to Fracture (2014) [11]	Education	Education on how to meet guideline recommendations	N/A	N/A	N/A^d^
Diabetes Canada (n.d.-b) [85]	Physical Activity and Diabetes (2018) [2]	Education	Education on how to meet guideline recommendations via introductory-level resistance training	N/A	N/A	N/A^d^
Diabetes Canada (n.d.-c) [86]	Physical Activity and Diabetes (2018) [2]	Education	Education on how to meet guideline recommendations via aerobic exercise	N/A	N/A	N/A^d^
Diabetes Canada (n.d.-e) [87]	Physical Activity and Diabetes (2018) [2]	Education	Education on walking	N/A	N/A	N/A^d^
Diabetes Canada (n.d.-f) [88]	Physical Activity and Diabetes (2018) [2]	Education	Education on resistance training	N/A	N/A	N/A^d^

^*^FAQ Frequently Asked Questions, *HCP* Healthcare Professionals, *MS* Multiple Sclerosis, *NS* Non-significant, *PA* Physical Activity, *SCI* Spinal Cord Injury

^a^ in more than 1 strategy category

^b^ Experimental

^c^ Observational

^d^ N/A

#### RQ3: Of the dissemination strategies evaluated, which were reported to be effective in enhancing guideline awareness, attitudes, and knowledge?

Over the two evaluated dissemination process strategies, both cross-sectional studies involving “formative research” demonstrated that physicians and end users had positive perceptions of the appropriateness, utility, and clarity of guideline messages [40, 41]. Of the four evaluated dissemination strategies, one cross-sectional study [52] involving “distribution of guideline materials” found low levels of guideline awareness (23.7%) prior to distribution of materials, but omitted a follow-up assessment. One prospective study [90] found “education” to be associated with significant increases in guideline awareness and knowledge among healthcare professionals and trainees, with increases in awareness being sustained at one-month follow-up. However, this study found that low levels of guideline awareness (17.8%) and knowledge (4.3%) among practicing healthcare professionals persisted across time-points [90]. Finally, one RCT [89] found “education” to be associated with significant increases in attitudes toward the guidelines among adults with MS from pre- to post-intervention; increases were not maintained at three-week follow-up.

### Implementation

#### RQ4: What strategies have been used in Canada to implement PA guidelines for specific populations?

Thirty-nine records reported on one or more implementation strategies (i.e., 8 records used 2 strategies [97, 98, 100–102, 106–108]; 2 records used 4 strategies [42, 103] for a total of 53 instances of the use of an implementation strategy). All four implementation strategy categories were represented, with integration strategies as the most commonly used category (*n* = 31), followed by implementation process (*n* = 9), scale-up (*n* = 7), and capacity-building (*n* = 6) strategies.

Five of the nine implementation process strategies were categorized as “engaging stakeholders”. For example, Gainforth et al.[97] partnered with three organizations to co-develop a workshop to promote the 2011 SCI guidelines [53]. The other four implementation process strategies were categorized as “human resources”. For instance, Parkinson Canada [100] employed studio directors to deliver a dance program to help adults with Parkinson’s disease engage in guideline-level PA.

Regarding the 31 integration strategies identified, six types of strategies were used. “Feedback” was used in one instance, where Trinh et al. [103] provided participants with activity trackers to view real-time feedback of their daily PA levels. Interestingly, this study also utilized “alerts”, “financial incentives”, and “planning tools” [103]. Worn activity trackers also gave sensory alerts to break up sedentary time lasting 30 min or longer to help participants meet the 2015 guidelines for people with cancer [6]. Participants were awarded points for engaging with the intervention that could be redeemed for items valuing a maximum of $50 CAD. Next, there were five instances of “skills training”. For example, Santa Mina et al. [102] had qualified professionals deliver an in-person 30-week exercise program to help adults with cancer meet the PA guidelines. Seven instances of “counselling” were identified. For example, Tomasone et al. [108] used telephone-based counselling to promote PA in line with the guidelines among clients with SCI. Lastly, “planning tools” were utilized in 16 instances. For instance, McMaster University’s Physical Activity Centre of Excellence [111] offers the “MS Get Fit Toolkit Online”, an interactive e-learning module for adults with MS in achieving guideline-recommended PA.

All six capacity-building strategies involved “stakeholder training”. In one, Salci et al. [107] delivered an online mentorship program to train adults with SCI and able-bodied personal trainers to enhance their self-efficacy to promote PA to adults with SCI. All seven instances of scale-up strategies used “implementation toolkits”. For example, CSEP’s [122] PARmed-X tool is available on their website for any healthcare provider to use when assessing whether patients who are pregnant may safely engage in PA in accordance with the pregnancy guidelines [1].

#### RQ5: Of the implementation strategies used, how have they been evaluated?

Out of 53 instances of an implementation strategy, 23 (43%) were evaluated [i.e., 2 implementation process strategies, 16 integration strategies (1 “feedback”, 1 “alerts”, 1 “financial incentives”, 4 “skills training”, 6 “counselling”, 3 “planning tools”) and 5 capacity-building strategies]. No scale-up strategies were evaluated. Five records evaluated multiple implementation strategies, totaling 16 records evaluating 23 implementation strategies.

Evaluated outcomes included self-efficacy [42, 98, 109, 110] and intention to meet guideline recommendations [42, 106, 108, 109], and behaviour in line with guideline recommendations [42, 98, 101–103, 108–110]. Included studies reported self-efficacy through subjective measures (i.e., task self-efficacy questionnaire items [98]; social cognitive predictors of Leisure Time PA (LTPA) among adults with SCI [109]; perceived capability to increase aerobic exercise) [110]. Intention was measured subjectively (i.e., intention to be active [42]; Health Action Process Approach stages of change [106]; LTPA intentions [108]; social cognitive predictors of LTPA among adults with SCI) [109]. Lastly, behaviour was assessed subjectively (i.e., LTPAQ-SCI [42, 98, 106, 108, 109]; GLTEQ-leisure score [102, 110]; International PA Questionnaire) [101] and objectively (i.e., Jawbone activity trackers [103]; wrist accelerometers) [98].

Implementation process outcomes included the quality of completion of activities related to implementation process strategies [97, 130]. Implementation capacity-building outcomes included self-efficacy to engage in implementation process strategies [97, 107, 117–119]. Outcomes beyond those specified in RQ6 are reported in Table 5.

**Table 5:** Implementation strategies identified

Record	Guideline	RQ1: Strategy	RQ2: Evaluation	RQ3: Outcomes	ROB	Record
		**Type**	**Description**			
**IMPLEMENTATION PROCESS STRATEGIES (*n* = 9)**						
^a^Gainforth et al. (2015) [97]	SCI Exercise Guidelines (2011) [8]	Engaging stakeholders	Partnership created between 3 groups (SCI Action Canada, Centre for Collaboration, Motivation, and Innovation, and SCI Ontario) to develop the Brief Action Planning (BAP) training workshop to promote PA to people with SCI	One arm pre-post using interviews (narrative analysis) and surveys (training satisfaction)	Perceived behavioural control to use BAP increased from baseline to post intervention but was not maintained at follow up (*p* > 0.05). 1 participant reached the level of competence appropriate to be certified to use BAP. Satisfaction with training was positive (all scored between 5–7/10)	Serious^c^
Latimer-Cheung (n.d.) [130]	Canadian Physical Activity Guidelines for Adults with MS (2013) [10]	Engaging stakeholders	Consumer and HCP feedback on the developed MS PA toolkit	Cross-sectional survey	HCP indicated they would use the toolkit in their practice if given the opportunity (mean score 5.45/10 (± 1.214); consumers indicated the toolkit will motivate (mean score 5.26/10 (± 1.298)) and teach (5.53/10 (± 1.379)) adults with MS how to make smart and informed decisions about PA and felt the guideline well-described the types of activities that can be used to meet guidelines (5.86/10 (± 1.159)); HCP and consumers felt strategies to overcome common barriers to exercise were appropriate (HCP mean 5.64/10 ± 1.027; consumer mean 5.14/0 ± 1.549), and all felt the tool was clear (consumer mean 5.92/10 + / 1.251; HCP mean 5.45 ± 1.695) and comprehensive (consumer mean 5.64/10 ± 1.222; HCP mean 5.27/10 ± 0.786)	Serious^d^
Clark et al. (2017) [52]	Too Fit to Fracture (2014)[11]	Engaging stakeholders	Physicians were interviewed to understand their thoughts, acceptability/usability, current practices, prior knowledge, barriers to using them in practice and what would be needed to use implement the recommendation use	N/A	N/A	N/A^e^
^a^Ma et al. (2019) [98]	Scientific Exercise Guidelines for Adults with SCI (2017) [9]	Engaging stakeholders	Stakeholders (physiotherapists and clients with SCI) were engaged to co-develop an RCT (introductory personal training session followed by eight weekly 15-min PA behavioral coaching sessions per week)	N/A	N/A	N/A^e^
Shirazipour (2013) [43]	Canadian Physical Activity Guidelines for Adults with MS (2013) [10]	Engaging stakeholders	Meeting minutes from consensus panel to develop and disseminate an evidence-based toolkit to inform adults of the guidelines and teach them to make smart, informed choices about PA and goal setting (topics included format and use of photos, etc.)	N/A	N/A	N/A^e^
^a^Santa Mina et al. (2019) [101]	Exercise for People with Cancer (2015) [6]	Human resources	Registered kinesiologists deliver a hospital-based exercise program	N/A	N/A	N/A^e^
^a^Santa Mina et al. (2017) [102]	Exercise for People with Cancer (2015) [6]	Human resources	Qualified professionals (physiotherapists, kinesiologists, and exercise physiologists) offer a 30 week community based exercise program	N/A	N/A	N/A^e^
^a^Latimer-Cheung et al. (2013) [42]	SCI Exercise Guidelines (2011) [8]	Human resources	A certified personal trainer and a peer co-delivered home-based strength training sessions	N/A	N/A	N/A^e^
^a^Parkinson Canada (n.d.-b)[100]	Physical Activity and Parkinson’s Disease (2012) [12]	Human resources	Exercise program (“Dancing with Parkinson’s”) with weekly dance class delivered by studio directors at a local dance school for people with Parkinson’s disease	N/A	N/A	N/A^e^
**INTEGRATION STRATEGIES (*n* = 31)**						
^a^Trinh et al. (2018) [103]	Exercise for People with Cancer (2015) [6]	Feedback	Participants wore activity trackers that provided information on their daily step count and overall progress	Pre-post intervention using Jawbone activity tracker and surveys (Functional Assessment of Cancer Therapy – General)	Significant reduction in sedentary time (455.4 min/week) and increase in MVPA (44.1 min/week) at post treatment but NS differences from baseline to 12 week follow up. NS changes in light intensity PA. Significant increase in step count from baseline to post intervention (1535 average step increase). At phase 1, 59% of participants met target step count (1000 above baseline) but by phase 3 only 39% met the rising target (3000 above baseline). Significant improvements seen in emotional wellbeing (average 1.2 point increase on a scale from 0–28)	Serious^c^
^a^Trinh et al. (2018) [103]	Exercise for People with Cancer (2015) [6]	Alerts	Participants wore activity trackers that provided sensory alerts to stand after ≥ 30 min of sedentary time	Pre-post intervention using Jawbone activity tracker and surveys (functional assessment of cancer therapy – general)	Significant reduction in sedentary time (455.4 min/week) and increase in MVPA (44.1 min/week) at post treatment but NS differences from baseline to 12 week follow up. NS changes in light intensity PA. Significant increase in step count from baseline to post intervention (1535 average step increase). At phase 1, 59% of participants met target step count (1000 above baseline) but by phase 3 only 39% met the rising target (3000 above baseline). Significant improvements seen in emotional wellbeing (average 1.2 point increase on a scale from 0–28)	Serious^c^
^a^Trinh et al. (2018) [103]	Exercise for People with Cancer (2015) [6]	Financial incentives	Participants were awarded points to encourage engagement with the intervention, which could be redeemed for a maximum of $50 CAD	Pre-post intervention using Jawbone activity tracker and surveys (functional assessment of cancer therapy – general)	Significant reduction in sedentary time (455.4 min/week) and increase in MVPA (44.1 min/week) at post treatment but NS differences from baseline to 12 week follow up. NS changes in light intensity PA. Significant increase in step count from baseline to post intervention (1535 average step increase). At phase 1, 59% of participants met target step count (1000 above baseline) but by phase 3 only 39% met the rising target (3000 above baseline). Significant improvements seen in emotional wellbeing (average 1.2 point increase on a scale from 0–28)	Serious^c^
^a^Santa Mina et al. (2019) [101]	Exercise for People with Cancer (2015) [6]	Skills training	Hospital based exercise program delivered by registered kinesiologists with prescribed at home component	Prospective cohort using surveys (IPAQ, SF-36), 6 min walk test	All participants were meeting PA guidelines at all time-points of the intervention. Improvements in the 6 min walk test from baseline to 48 weeks were seen (+ 72 m). Improvements in SF36 measures of general health (+ 4.4 points) and physical (+ 3.6 points) were seen at 48 weeks	Serious^c^
^a^Santa Mina et al. (2017) [102]	Exercise for People with Cancer (2015) [6]	Skills training	Qualified professionals (physiotherapists, kinesiologists, and exercise physiologists) offering a 30 week community based exercise program	Prospective cohort using surveys (GLTEQ, FACIT-F and G, self-rated health), 6 min walk test, grip strength, HR, BP, BMI, WC and functional reach	NS increase in MVPA minutes/week (+ 47); significant increase in LTPA from baseline to 10 weeks; significant increase in MET hours/week (4.46). Statistically significant increase from baseline to 10 weeks in fatigue, social wellbeing, 6 min walk test, RHR, SBP and functional reach	Serious^c^
^a^Latimer-Cheung et al. (2013) [42]	SCI Exercise Guidelines (2011) [8]	Skills training	Study 2/2: One home-based strength training session co-delivered by a certified personal trainer and a peer, paired with a 1-week action plan	One arm pre-post intervention using surveys (intention to be active, modified LTPAQ-SCI, health care climate questionnaire)	Significant increase in intentions to be active (*d* = 0.6) and action planning (*d* = -1.14) over the intervention. Significant increase in number of bouts of strength training, duration, and total min/week at the 4 week follow up (*p* < 0.024). Significant increase in task frequency self efficacy and barrier self-efficacy (*d* = 0.52; *d* = 0.87)	Serious^c^
^a^Ma et al. (2019) [98]	Scientific Exercise Guidelines for Adults with SCI (2017) [9]	Skills training	Introductory personal training session followed by 8 behavioural coaching sessions delivered by an exercise professional (weekly 15-min sessions)	RCT using surveys (LTPAQ-SCI, measures of health action process, self-efficacy), wrist accelerometer	Increases in all but 1 participant in MVPA from baseline to post-intervention (+ 236 min/week); larger effect size when self-reported vs when looking at accelerometer. Increase in total LTPA from baseline to 6 month follow up (+ 348 min/week). Increase in task self-efficacy for all participants for engaging in strength exercise, with a significantly greater increase in intervention vs control group (*p* < 0.05)	High^b^
^a^Parkinson Canada (n.d.-b)[100]	Physical Activity and Parkinson’s Disease (2012) [12]	Skills training	Exercise program (“Dance with Parkinson’s”) with weekly dance class delivered by studio directors at a local dance school for people with Parkinson’s disease	N/A	N/A	N/A^e^
Vallerand et al. (2019) [110]	Exercise for People with Cancer (2015) [6]	Counselling	12-week theory-based telephone counselling (1 session/week) intervention to encourage cancer survivors to increase their PA by 60 min/week	RCT using surveys (affective attitude, action planning, instrumental attitude, modified GLTEQ, perceived capability and opportunity)	Significantly greater increases in instrumental attitude in intervention group vs control (MBGD = 0.5). Small between group difference noted for action planning (MBGD = 0.3) and medium between group differences in affective attitude were observed (MBGD = 0.06). Intervention group increased exercise behaviour by 281 min (versus 93 min in the control). Small between group differences (MBGD = 0.2) at the end of intervention in perceived capability to increase aerobic exercise and medium between group difference seen in having the opportunity to increase weekly aerobic exercise (MBGD = 0.4)	High^b^
^a^Arbour-Nicitopoulos et al. (2014) [106]	SCI Exercise Guidelines (2011) [8]	Counselling	6 month telephone counselling with trained counsellor delivered with a Welcome Package (resistance bands, an instruction guide, tip sheets, and goal-achievement strategies) to develop and strengthen social cognitions for engaging in LTPA	Prospective cohort using surveys (LTPA intentions, LTPAQ-SCI)	Intentions to engage in LTPA started high (average 4.54/7) and remained high (*p* = 0.44). More clients engaged in moderate-high intensity LTPA at 6 months vs baseline (*p* = 0.09). NS increase in % of clients regularly active at baseline vs 4 (*p* = 0.13) or 6 (*p* = 0.09) months	Serious^c^
Chemtob et al. (2019) [109]	Scientific Exercise Guidelines for Adults with SCI (2017) [9]	Counselling	One counselling session per week for 8 weeks with a trained registered kinesiologist to motivate participants to engage in LTPA	RCT using surveys (treatment self-regulation questionnaire, LTPAQ-SCI, series assessing social cognitive predictors)	Intervention group reported greater autonomous motivation (Hedge’s g = 0.91) and controlled motivation at 6 (Hedges' g = − 0.24) and 10 weeks (Hedges' g = 0.02). They reported greater total LTPA at 6 (Hedges' g = 0.87) and 10 (Hedge’s g = 0.85) weeks and showed a greater change from baseline to 6 (dppc = 1.14) and 10 (dppc = 1.28) weeks	High^b^
^a^Latimer-Cheung et al. (2013)[42]	SCI Exercise Guidelines (2011) [8]	Counselling	Study 1/2: One 30 min telephone-based counselling session on self-regulation and action plans	One arm pre-post intervention using surveys (intention to be active, modified LTPAQ-SCI, health care climate questionnaire)	Significant increase in intentions to be active (*d* = 0.6) and action planning (*d* = -1.14) over the intervention. Significant increase in number of bouts of strength training, duration, and total min/week at the 4 week follow up (*p* < 0.024). Significant increase in task frequency self efficacy and barrier self-efficacy (*d* = 0.52; *d* = 0.87)	Serious^c^
^a^Tomasone et al. (2018) [108]	SCI Exercise Guidelines (2011) [8]	Counselling	Telephone-based counselling by a registered kinesiologist paired with the SCI Get Fit Toolkit, gradually progressing to self-regulation (i.e., counselling delivered weekly for months 1 & 2, biweekly for months 3 & 4, and monthly for 5 & 6). Intervention materials included: resistance bands, an instruction guide, tip sheets, and an activity intensity classification chart	One arm pre-post intervention using surveys (LTPA intentions, LTPAQ-SCI	Baseline intentions for engaging in aerobic, strength, and total LTPA were high (6.14/7 ± 1.44) and did not change over the 6 month intervention. Significant effect in time spent strength training and total MVPA over the intervention (all F(2,40) *s* ≥ 3.679, ps = 0.03). NS change in aerobic activity over the intervention though small increases emerged between baseline and 2 months (*d* = 0.29), and baseline and 6 months (*d* = 0.2). Clients had positive perceptions of the information and resources provided (all M*s* ≥ 6.00 out of 7)	Serious^c^
^a^Ma et al. (2019) [98]	Scientific Exercise Guidelines for Adults with SCI (2017) [9]	Counselling	Behavioural coaching sessions and personal training by professionals (8 weekly 15-min sessions)	RCT using surveys (LTPAQ-SCI, measures of health action process, self-efficacy), wrist accelerometer	Increases in all but 1 participant in MVPA from baseline to post-intervention (+ 236 min/week); larger effect size when self-reported vs when looking at accelerometer. Increase in total LTPA from baseline to 6 month follow up (+ 348 min/week). Increase in task self-efficacy for all participants for engaging in strength exercise, with a significantly greater increase in intervention vs control group (*p* < 0.05)	High^b^
^a^Salci et al. (2016) [107]	SCI Exercise Guidelines (2011) [8]	Counselling	Adults with SCI and exercise professionals trained by the ALLTP online mentorship program offered counselling to promote LTPA to people with SCI	N/A	N/A	N/A^e^
^a^Trinh et al. (2018) [103]	Exercise for People with Cancer (2015) [6]	Planning tools	Participants were granted access to online action planning resources to help them be more active	Pre-post intervention using Jawbone activity tracker and surveys (functional assessment of cancer therapy – general)	Significant reduction in sedentary time (455.4 min/week) and increase in MVPA (44.1 min/week) at post treatment but NS differences from baseline to 12 week follow up. NS changes in light intensity PA. Significant increase in step count from baseline to post intervention (1535 average step increase). At phase 1, 59% of participants met target step count (1000 above baseline) but by phase 3 only 39% met the rising target (3000 above baseline). Significant improvements seen in emotional wellbeing (average 1.2 point increase on a scale from 0–28)	Serious^c^
^a^Arbour-Nicitopoulos et al. (2014) [106]	SCI Exercise Guidelines (2011) [8]	Planning tools	6 month telephone counselling with trained counsellor delivered with a Welcome Package (resistance bands, an instruction guide, tip sheets, and goal-achievement strategies) to develop and strengthen social cognitions for engaging in LTPA	Prospective cohort using surveys (LTPA intentions, LTPAQ-SCI)	Intentions to engage in LTPA started high (average 4.54/7) and remained high (*p* = 0.44). More clients engaged in moderate-high intensity LTPA at 6 months vs baseline (*p* = 0.09). NS increase in % of clients regularly active at baseline vs 4 (*p* = 0.13) or 6 (*p* = 0.09) months	Serious^c^
^a^Latimer-Cheung et al. (2013) [42]	SCI Exercise Guidelines (2011) [8]	Planning tools	One home based strength training session co-delivered by a certified personal trainer and a peer, paired with a 1-week action plan	One arm pre-post intervention using surveys (intention to be active, modified LTPAQ-SCI, health care climate questionnaire)	Significant increase in intentions to be active (*d* = 0.6) and action planning (*d* = -1.14) over the intervention. Significant increase in number of bouts of strength training, duration, and total min/week at the 4 week follow up (*p* < 0.024). Significant increase in task frequency self efficacy and barrier self-efficacy (*d* = 0.52; *d* = 0.87)	Serious^c^
Cancer Care Ontario (n.d.-d)[114]	Exercise for People with Cancer (2015) [6]	Planning tools	Handout sheet for setting PA goals	N/A	N/A	N/A^e^
SCI Action Canada (2011c)[54]	SCI Exercise Guidelines (2011) [8]	Planning tools	SCI Get Fit Toolkit brochure with sample activity plan	N/A	N/A	N/A^e^
SCI Action Canada (n.d.-b)[93]	SCI Exercise Guidelines (2011) [8]	Planning tools	Home strength training guide with planning worksheets	N/A	N/A	N/A^e^
McMaster University (n.d.) [111]	Canadian Physical Activity Guidelines for Adults with MS (2013) [10]	Planning tools	Interactive e-learning module (MS Get Fit Toolkit Online) to provide practical information on how to achieve MS guideline level activity	N/A	N/A	N/A^e^
MS Society of Canada (n.d.-a)[113]	Canadian Physical Activity Guidelines for Adults with MS (2013) [10]	Planning tools	Sub-portion of the MS Get Fit Toolkit including a goal setting template to help people with MS set and achieve exercise goals	N/A	N/A	N/A^e^
MS Society of Canada (n.d.-b)[69]	Canadian Physical Activity Guidelines for Adults with MS (2013) [10]	Planning tools	MS Get Fit Toolkit: sample exercises for different movement levels to help people with MS meet each guideline component and provides common exercise barriers and strategies to overcome them	N/A	N/A	N/A^e^
MS Society of Canada (n.d.-c)[112]	Canadian Physical Activity Guidelines for Adults with MS (2013) [10]	Planning tools	Website containing various resources (toolkit, handouts, guide) for planning exercise, basic exercises and a guide for people to follow if they wish to engage in more activity	N/A	N/A	N/A^e^
Osteoporosis Canada (n.d.-b)[75]	Too Fit to Fracture (2014)[11]	Planning tools	Booklet containing sample exercises, activity planning worksheets, and guide for how to achieve each recommendation (i.e., how to get 30 min aerobic)	N/A	N/A	N/A^e^
Parkinson Society Canada (2012) [12]	Physical Activity and Parkinson’s Disease (2012) [12]	Planning tools	PA progress chart to note daily activity type and duration for one month	N/A	N/A	N/A^e^
Diabetes Canada (n.d.-a) [49]	Physical Activity and Diabetes (2018) [2]	Planning tools	Brochure with a motivation checklist and an interactive exercise to help end-users identify personal barriers to PA and can consider strategies to overcome common barriers	N/A	N/A	N/A^e^
Diabetes Canada (n.d.-c) [86]	Physical Activity and Diabetes (2018) [2]	Planning tools	Brochure explaining how to set SMART (specific, measureable, attainable, realistic, time-oriented) exercise goals, and how to create and maintain an aerobic training program	N/A	N/A	N/A^e^
Diabetes Canada (n.d.-e)[87]	Physical Activity and Diabetes (2018) [2]	Planning tools	Brochure with an activity sheet for considering personal pros and cons of being active/inactive and a template for creating a weekly PA plan	N/A	N/A	N/A^e^
Ontario Brain Institute (2014b) [115]	Formulation of evidence-based messages to promote the use of physical activity to prevent and manage Alzheimer’s disease (2017) [3]	Planning tools	Tracking sheet to write down sources of personal motivation and goals, and to build and plan how to maintain a weekly PA plan	N/A	N/A	N/A^e^
**CAPACITY-BUILDING (*n* = 6)**						
Tomasone et al. (2017) [119]	SCI Exercise Guidelines (2011) [8]	Stakeholder training	Seminars provided to HCP trainees during regular class time to teach how to discuss LTPA among their patients with SCI	One arm pre-post intervention using surveys	Significant linear increase immediately post training in belief that attending a presentation will help discuss PA to future patients (5.62 to 6.2, out of 7), followed by linear decrease over subsequent 6 months (5.59) (*αs* ≥ .81). Confidence in ability to discuss PA with future patients followed the same trend (4.91 to 5.84 to 5.25; *rs* ≥ .71). Inclusion of audiovisual presentation aspects predicted positive changes in attitudes pre/post intervention (*p* < 0.001)	Serious^c^
Tomasone et al. (2015) [118]	SCI Exercise Guidelines (2011) [8]	Stakeholder training	Training sessions for CMCL presenters (i.e., facilitators) how to run the CMCL intervention for people with SCI either face to face or via telephone (the CMCL intervention aims to increase HCPs use of PA guidelines for people with SCI)	One arm pre-post intervention using surveys	NS changes at any time-point in perceptions that the new CMCL curriculum will help presenters implement the CMCL info, strategies, and resources at their next presentation (pre score: 5.89/7). NS changes in % of the new CMCL curriculum presenters intend to use at their next presentation. Significant decrease from post intervention to 6 month follow up in confidence in ability to tell HCP about CMCL info, strategies and resources, persuade HCP to use CMCL resources, teach presenters about CMCL and persuade presenters to use CMCL information (6.2/7 fell to 5.65/7; effect size -0.77)	Serious^c^
Tomasone et al. (2014) [117]	SCI Exercise Guidelines (2011) [8]	Stakeholder training	CMCL seminars delivered to HCPs (e.g., rehabilitation therapists) to enhance their intentions to prescribe PA to patients	One arm pre-post intervention using surveys (self efficacy items adapted from Rhodes and Courneya, intention items adapted from Azjen)	Intentions to discuss PA significantly increased from pre-to-post CMCL training (*p* < 0.002) but significant decreases were seen between post training and 6 months *(p* < 0.005) (no decline below baseline). Same trend was seen in confidence in their ability to discuss PA with patients and persuade patients to participate in PA, and in instrumental attitudes towards the usefulness of CMCL	Serious^c^
^a^Gainforth et al. (2015) [97]	SCI Exercise Guidelines (2011) [8]	Stakeholder training	One 4-h workshop offered in 3 regional areas by a certified Brief Action Planning (BAP) trainer to teach peers BAP and motivational interviewing to promote PA to people with SCI	One arm pre-post intervention using interviews (narrative analysis) and surveys (training satisfaction)	Perceived behavioural control to use brief action planning increased from baseline to post intervention but was not maintained at follow up (*p* > 0.05). 1 participant reached the level of competence appropriate to be certified to use BAP. Satisfaction with training was positive (all scored ranging from 5–7/10)	Serious^c^
^a^Salci et al. (2016) [107]	SCI Exercise Guidelines (2011) [8]	Stakeholder training	HCP completed the ALLTP program to help them encourage and recommend PA to patients with SCI	Quasi-experimental pre/post intervention using surveys	The ALLTP module left participants feeling their self-efficacy was enhanced to speak about and encourage LTPA. It remained high throughout training and positively correlated with the usefulness of program content (*r* = 0.41–0.71). At follow up, participants had discussed LTPA with an average of 7 people with disabilities	Serious^c^
Schmitz et al. (2019) [120]	Exercise Guidelines for Cancer Survivors: Consensus Statement from International Multidisciplinary Roundtable (2019) [7]	Stakeholder training	Suggestions for HCP to work toward implementing the guidelines in their practice	N/A	N/A	N/A^e^
**SCALE-UP (*n* = 7)**						
Oncology Nursing Society (n.d.) [123]	Exercise for People with Cancer (2015) [6]	Implementation toolkit	Checklist can be used by any HCP to help recommend PA to cancer patients and identify risk factors	N/A	N/A	N/A^e^
Exercise is Medicine (n.d.-b) [124]	Exercise Guidelines for Cancer Survivors: Consensus Statement from International Multidisciplinary Roundtable (2019) [7]	Implementation toolkit	Online program registry to help patients, families, and HCP find PA programs in their communities	N/A	N/A	N/A^e^
Exercise is Medicine (n.d.-c) [125]	Exercise Guidelines for Cancer Survivors: Consensus Statement from International Multidisciplinary Roundtable (2019) [7]	Implementation toolkit	Fillable handout for HCPs to help make PA recommendations (prescriptions) to patients (including dose, type, etc. for aerobic and strength training)	N/A	N/A	N/A^e^
Ma et al. (2018) [92]	SCI Exercise Guidelines (2011) [8]	Implementation toolkit	Tool helps HCP recommend PA to patients with SCI depending on risk factors, motivation, and resource availability	N/A	N/A	N/A^e^
SCI Action Canada (n.d.-b) [127]	SCI Exercise Guidelines (2011) [8]	Implementation toolkit	Series of online videos to provide HCPs with the latest knowledge, resources, barriers people with SCI face to PA, coping strategies, and tips to lead patients to be more active	N/A	N/A	N/A^e^
Canadian Society for Exercise Physiology (2015) [122]	Canadian Guideline for Physical Activity Throughout Pregnancy (2019) [1]	Implementation toolkit	Screening tool to help HCPs determine if their pregnant patients are ready to safely engage in PA	N/A	N/A	N/A^e^
Diabetes Canada (n.d.-d) [126]	Physical Activity and Diabetes (2018) [2]	Implementation toolkit	Interactive decision-making tool for HCP to easily recommend PA to patients with diabetes	N/A	N/A	N/A^e^

^*^ALLTP Active Living Leaders Training Program, *BMI* Body Mass Index, *BP* Blood Pressure, *CMCL* Changing Minds Changing Lives, *CSEP* Canadian Society for Exercise Physiology, *FACIT-F* Functional Assessment of Chronic Illness Therapy – Fatigue, *GLTEQ* Godin Leisure Time Exercise Questionnaire, *HCP* Healthcare Professionals, *IPAQ* International Physical Activity Questionnaire, *LTPA* Leisure Time Physical Activity, *LTPAQ-SCI* Leisure Time Physical Activity Questionnaire – Spinal Cord Injury, *MBGD* Mean Between Group Difference, *MET* Metabolic Equivalent, *MS* Multiple Sclerosis, *MVPA* Moderate to Vigorous Physical Activity, *NS* Non-significant, *PA* Physical Activity, *RHR* Resting Heart Rate, *SCI* Spinal Cord Injury, *SF-36* Short form 36, *WC* Waist Circumference

^a^ in more than 1 strategy category

^b^ RCT

^c^ Experimental

^d^ Observational

^e^ unclear

#### RQ6: Of the implementation strategies evaluated, which were reported to be effective for enhancing self-efficacy, intention, and behaviour in line with the guideline, and self-efficacy and intent to use the guideline?

In the two evaluated implementation process strategies, “engaging stakeholders” was associated with increased social cognitions to perform an integration strategy among trainers in one experimental study [97] and among healthcare professionals in one cross-sectional study [130].

Of the 16 integration strategies evaluated, “feedback” was associated with significant increases in PA in line with the guideline in one experimental study [103]. “Skills training” was evaluated in four experimental studies, reporting increases in self-efficacy [98], intentions [42], and PA behaviour [42, 98, 102]. One of these studies found no significant changes, but identified a high adherence to guideline-level PA behaviour across all time-points [101]. “Counselling” was evaluated in six experimental studies, showing positive associations with self-efficacy [98, 109, 110], intentions [42, 106, 108, 109], and PA behaviour in line with the guidelines [42, 98, 108, 110]. Three studies found “planning tools” to be associated with significant increases in intentions [42, 106] and guideline-level PA [42, 103]. Interestingly, Trinh et al., [103] who incorporated “feedback”, “alerts”, “financial incentives” and “planning tools”, found significant increases in PA in line with the guidelines.

Latimer-Cheung et al. [42] also amalgamated four strategies; however, this involved three integration strategies (i.e., “skills training”, “counselling”, and “planning tools”) and one implementation process strategy (i.e., “human resources”). Two studies combined two integration strategies (i.e., “counselling” and “planning tools” [106]; “skills training” and “counselling”) [98], and four studies combined one integration strategy with either one implementation process strategy or one capacity-building strategy (i.e., “counselling” with “human resources” [100–102] or with “stakeholder training”) [107]. Given their study designs, we cannot determine whether positive outcomes resulted from a single implementation strategy, or from multiple, concurrent implementation strategies.

Of the five evaluated capacity-building strategies, “stakeholder training” was associated with high levels of self-efficacy to engage in the implementation process strategy across five experimental studies [97, 107, 117–119]. Logically, one of these studies paired “engaging stakeholders”, an implementation process strategy, with “stakeholder training” to promote PA in adults with SCI [97].

### Risk of Bias

Of the six records evaluating six dissemination strategies, all but one used a non-randomized study design and were rated as “serious” ROB (see last column in Table 4) because of missing data (*n* = 3) [40, 41, 90], subjective outcome measurement (*n* = 1) [90], and confounding bias (*n* = 1) [90]. The one RCT was rated as “high” ROB (see last column in Table 4) due to blinding participants and assessments, attrition bias, and large initial between-group differences [89].

Of the 16 records that evaluated 23 evaluated implementation strategies, 13 were non-randomized study designs and were all deemed as “serious” ROB (see last column in Table 5) due to confounding bias (*n *= 11) [42, 97, 101–103, 106–108, 117–119], sampling (*n *= 4) [97, 102, 103, 107], intervention measurement (*n *= 4) [42, 102, 106, 108], outcome measurement (*n *= 6) [42, 97, 101, 102, 106, 117], and missing data (*n *= 1) [130].

The remaining three records evaluating implementation strategies were all RCTs deemed as “high” ROB (see last column in Table 5) due to lack of blinding participants and assessments (*n* = 3) [98, 109, 110] and attrition bias (*n *= 2) [98, 109]. Full ROB ratings can be seen in Supplement 2 (RCTs) and Supplement 3 (non-randomized, quasi-experimental, and observational study designs).

## Discussion

This systematic scoping review aimed to identify and evaluate strategies used for the D&I of PA guidelines among adults of specific populations and/or their stakeholders in Canada. This review adds to Tomasone et al.’s [23] findings as we discovered new strategy types, identified evaluations of capacity-building strategies, and found more concurrent uses of D&I strategies, which may help guide efforts to translate guidelines into use across both general and specific populations, within Canada or internationally.

### Dissemination

Six dissemination process strategies involved “formative research” to support the dissemination of two guidelines (i.e. for MS and Alzheimer’s) [3, 10]. Further, we identified 49 dissemination strategies – a greater number than identified in Tomasone et al.’s [23] review of guideline D&I for the general population. “Distribution of guideline materials” was the most commonly used dissemination strategy type (*n* = 30), while “mass media/communications campaigns” was the least common (*n *= 9). Only four records evaluated two types of dissemination strategies (i.e., “distribution of guideline materials” and “education”) and no included record evaluated “mass media/communications campaigns”. Many included records reported distributing fact sheets as a dissemination strategy, potentially due to their simplicity and low production cost relative to other dissemination strategies (e.g., scientific reports, mass media campaigns) [131]. While no included study evaluated a fact sheet, prior research has found fact sheets to improve Australian health professionals’ knowledge and intentions to advise against alcohol consumption during pregnancy [132] and to enhance North American and Western European women’s knowledge and attitudes about congenital infections as effectively as educational videos [133].

As the majority of dissemination strategies were not evaluated, it is difficult to reliably conclude how effective these strategies are at enhancing awareness, attitudes, and knowledge toward PA guidelines. Similarly, we cannot determine whether any of the evaluated strategies were more or less effective than others due to the heterogeneous evaluation measures used. Nevertheless, our findings align with Tomasone et al., [23] suggesting that low levels of guideline awareness and knowledge persisted despite the use of abovementioned strategies to disseminate PA guidelines. Formative research (e.g., engaging stakeholders) is warranted for future studies to identify how dissemination strategies may be best enacted in real-world settings and evaluation is warranted to determine which have the most utility for improving guideline awareness and knowledge among specific populations and their stakeholders.

### Implementation

Nearly as many implementation strategies were identified as dissemination strategies (*n* = 53 and *n* = 55, respectively). The most-used implementation strategy was “planning tools” (*n* = 16) with “feedback”, “alerts”, and “financial incentives” being the least-often used (*n*s = 1). Unique from Tomasone et al. [23] was our identification of multiple records discussing multiple implementation strategies for a single intervention (i.e., the Changing Minds, Changing Lives intervention promoted the uptake and use of the SCI guidelines among stakeholders) [90, 117–119]. The researchers of the Changing Minds, Changing Lives intervention evaluated “education” and “stakeholder training” strategies, which informed multiple intervention iterations. Possibly, sequentially applying strategies in a single intervention may provide greater opportunities to assess the effectiveness of, and thus improve upon, D&I efforts.

Nevertheless, few implementation strategies were evaluated and evaluations applied a variety of measures, particularly for self-efficacy. This makes cross-comparison a challenge, rendering conclusions on relative effectiveness impossible. Of the evaluated implementation strategies, “counselling” and “skills training” seem to have been most successful at enhancing self-efficacy, intention, and behaviour to meet PA guideline benchmarks among members of specific populations and “stakeholder training” seemed to have had an influence on stakeholders’ self-efficacy to engage in implementation process strategies. “Counselling”, “skills training”, and “stakeholder training” were often delivered in-person, which is likely to be resource intensive. In light of the COVID-19 pandemic, remote PA counselling and skills training may be a more viable option [134]. Recent work has found that online PA interventions are not only more feasible to implement broadly in absence of research funding, but may also be as effective as in-person formats [135]. Indeed, remote formats may be beneficial for specific populations in overcoming their unique barriers to engaging in PA such as concerns overs accessibility and transportation [109].

Finally, 16 planning tools were implemented among specific populations, such as fillable handouts and checklists to assist individuals in setting PA goals, but only three were evaluated. Planning has been identified as an important, effective step for health behaviour change among the general population [136]. While singular implementation strategies, such as planning tools, may be lower cost [136] and easier to deliver than multifaceted interventions (e.g., combined PA counselling and PA training) [137], evaluating such tools may not be feasible for guideline developers if funding is limited [138]. Still, research utilizing planning tools should aim to investigate their impact on specific population health behaviour when possible.

### Combined uses of D&I strategies

Eleven records used D&I strategies concurrently, more than was identified in Tomasone et al. [23] This may be because stakeholders of PA guideline D&I for specific populations (e.g., guideline developers, organization members) are more integrated with their target communities than stakeholders of general population PA guidelines. For instance, “formative research”, “distribution of guideline materials”, “counselling”, and “planning tools” were used for both the MS Get Fit Toolkit and the SCI Get Fit Toolkit [43, 54] to simultaneously disseminate and implement the guidelines. However, no record concurrently evaluated D&I outcomes [43, 52]. It would be helpful to understand the impacts of concurrent use of D&I strategies, such as how targeting PA guideline awareness first may augment increases PA behaviour in line with the guidelines. From a theoretical stance, behavioural determinants, such as awareness or knowledge, are antecedents to longer-term outcomes, such as PA [23, 139]. Accordingly, future work should evaluate concurrent uses of D&I strategies to clarify their interplay in promoting the uptake and use of guidelines.

### Implications for future reporting and research

Interestingly, many identified records pertained to the PA guidelines for persons living with and beyond cancer (*n* = 29), and for persons with SCI (*n* = 21). Comparatively, the osteoporosis guidelines [11] were reflected in three records. Organizations could benefit from enhanced communication amongst each other and with researchers to endorse strategies deemed to be most effective, which can help advance guideline uptake and use [140]. Indeed, our content expert consultation identified many unpublished and non-public records (*n* = 43), which may be hampering important advances in the field. Thus, we recommend that researchers in this area make all guideline D&I strategies available, such as with open science, to improve communication.

Despite the smaller scope of our review (i.e., smaller geographic scope, smaller-sized populations, English only, PA guidelines only), more records of specific population PA guidelines were identified (*n* = 81) than the Tomasone et al. [23] review of PA, sedentary behaviour, and sleep guidelines (*n *= 47). Perhaps, strategies in specific populations are developed and enacted more frequently because specific populations are more defined, connected with health professionals (e.g., oncologists), and likely to ask for guideline resources relative to the general population [141]. Moreover, PA guidelines for specific populations are often disseminated and implemented with dedicated research grant funding with expectations for end-of-grant knowledge translation efforts by guideline development groups through existing networks with end-users. Nevertheless, no firm conclusions can be drawn regarding whether the higher number of identified records in this review resulted from specific population PA guidelines having been developed and supported by special interest groups. The high variability in methods used to evaluate D&I strategies created a challenge for determining strategies’ relative effectiveness. Unfortunately, some strategy classes (i.e., implementation scale-up strategies) and some types (i.e., “mass media/communications campaign”, “human resources”) were not evaluated, similar to Tomasone et al., [23] and dissemination scale-up strategies were not represented at all in our review. Finally, despite the usefulness of D&I process strategies in establishing the needs of the target audience [23], only six identified implementation strategies in our review were either paired with “stakeholder engagement” (i.e., integration [98]; capacity-building) [97] or “human resources” [42, 100–102].

These findings bear practical and knowledge implications for future study and reporting in D&I. First, the present results may inform future guideline D&I work in Canada or analogous countries (i.e., high-income status, English-speaking). Specifically, it appears that “counselling”, “skills training”, and “planning tools” are deemed to be effective at enhancing self-efficacy, intentions, and PA behaviour in line with guidelines [42, 98, 101, 102, 106, 108–110] and “stakeholder training” is deemed to be effective at improving self-efficacy to perform implementation process strategies [97, 107, 117–119]. Further, limited evidence suggests that “formative research” may be useful in determining the quality of dissemination efforts [40, 41] and “education” may be effective at increasing awareness and knowledge of PA guidelines [90]. Thus, to promote PA among specific populations, it is recommended that “formative research” and “education” be used to enhance awareness and knowledge and “counselling”, “skills training”, and “planning tools” be used to enhance self-efficacy, intentions, and PA behaviour in line with the guidelines. Future research should continually investigate their effectiveness, to support refinement and positive evaluations of these strategies among the general population [23], and should begin to evaluate other types of strategies (e.g., “mass media/communications campaigns”, “implementation toolkits”) to determine which contribute to positive outcomes. In all populations, future studies should incorporate more formative research on strategy development and improvement and continue using integrated knowledge translation approaches that engage relevant stakeholder groups from project outset [142], which may improve the potency of future guideline D&I efforts [143]. Overall, there is a need for further use and investigation of the effectiveness of a wider range of D&I strategies for both the general population and specific populations.

### Strengths and limitations

This systematic scoping review has several notable strengths. Primarily, the methodology used was rigorous and adhered to published standards [30, 31, 33]. The comprehensive search strategy identified 81 relevant records reporting D&I strategies. The majority of records were located through the content expert (*n* = 44) and targeted web-based (*n* = 27) search approaches, demonstrating the appropriateness of the systematic scoping review methodology used. Secondly, this review applied the framework by Tomasone et al., [23] enabling the classification of six dissemination process strategies, which showcased the utility of collaborative, formative approaches for enhancing dissemination strategies. The present study also highlighted a need for greater frequency and consistency in the evaluation of D&I strategies and their outcomes. Moving forward, comparative evaluations could help determine whether certain strategies are more effective than others within a given population, or are more effective in one population than another. Finally, while this review identified strategies for disseminating and implementing PA guidelines to only eight specific populations, the present findings could apply to other researchers disseminating or implementing PA guidelines more broadly. Along with findings from Tomasone et al. [23], it appears that “feedback”, “financial incentives”, “counselling”, and “planning tools” may be effective for PA guideline implementation regardless of the population of interest.

This review is not without its limitations. Specifically, our inclusion criteria may have limited the number of relevant records identified. For example, because some of the included guidelines are international in scope, such as the 2017 SCI guidelines [9], it is possible that they could have been disseminated and/or implemented by other countries; thus, our review precluded inclusion of such records (c.f., [144]) despite them utilizing the same guideline. Second, our review provides a snapshot of D&I strategies for specific population PA guidelines at a specific point in time, which is subject to change as additional D&I efforts are made. However of interest, even the content experts who led guideline D&I were not aware of any additional, recent records, suggesting that the majority of D&I efforts happen soon after a guideline is released. Further, the few evaluated strategies and large variation in evaluation methods did not support a comprehensive evaluation of the relative effectiveness of D&I strategies. Finally, the ROB tools used may have been a limitation as they identified a high degree of bias in the included studies, when the problem may instead be systemic. For instance, large sample sizes and control groups may not be possible or ethical when conducting PA research among specific populations as PA opportunities for these individuals tend to be highly sought-after yet rare [145].

## Conclusion

Few reports of the D&I of PA guidelines for specific populations have evaluated the strategy(ies) used. Nonetheless, this review identified favourable strategies for the dissemination (i.e., “formative research”, “education”) and implementation (i.e., “counselling”, “skills training”, “planning tools”, “stakeholder training”) of population-specific PA guidelines. Future initiatives to develop and apply D&I strategies should be accompanied by evaluation of those strategies wherever feasible. Studies could also look to evaluate a greater range of strategies to determine their relative effectiveness. Ultimately, these results can help inform future D&I efforts to translate PA guideline recommendations into use among specific populations and their stakeholders, which may inform similar efforts among the general population.

## Supplementary Information

**Additional file 1:Supplement 1. **Search terms for guideline identification, database searches, and targeted web-based search strings. **Supplement 2.** Risk of bias assessment for included randomized controlled trials. **Supplement 3.** Risk of bias assessment for included non-randomized interventions.

## Data Availability

The datasets used and/or analysed during the current study are available from the corresponding author on reasonable request.

## References

[1] Canadian Society for Exercise Physiology (2019). 2019 Canadian Guideline for Physical Activity throughout Pregnancy.

[2] Diabetes Canada. Physical activity and diabetes. https://www.diabetes.ca/static/docs/physical-activity-and-diabetes.pdf. Published 2018.

[3] Martin Ginis KA, Heisz J, Spence JC (2017). Formulation of evidence-based messages to promote the use of physical activity to prevent and manage Alzheimer’s disease. BMC Public Health.

[4] Ross R, Chaput J-P, Giangregorio LM (2020). Canadian 24-Hour Movement Guidelines for adults aged 18–64 years and adults aged 65 years or older: an integration of physical activity, sedentary behaviour, and sleep. Appl Physiol Nutr Metab.

[5] Martin Ginis KA, West CR (2021). From guidelines to practice: development and implementation of disability-specific physical activity guidelines. Disabil Rehabil.

[6] Segal R, Zwaal C, Green E (2015). Exercise for People with Cancer.

[7] American College of Sports Medicine. Exercise guidelines for cancer survivors: Consensus statement from International Multidisciplinary Roundtable. https://www.acsm.org/read-research/newsroom/news-releases/news-detail/2019/10/16/expert-panel-cancer-treatment-plans-should-include-tailored-exercise-prescriptions. Published 2019. 10.1249/MSS.0000000000002116 PMC8576825 31626055

[8] SCI Action Canada. Physical activity guidelines for adults with spinal cord injury. https://www.csep.ca/CMFiles/Guidelines/specialpops/SCIPAGuidelinesClient.pdf. Published 2011.

[9] SCI Action Canada. Scientific exercise guidelines for adults with spinal cord injury. https://ok-sciguidelines.sites.olt.ubc.ca/. Published 2017.

[10] Canadian Society for Exercise Physiology (2013). Canadian Physical Activity Guidelines for Adults with Multiple Sclerosis.

[11] Osteoporosis Canada. Too fit to fracture. https://osteoporosis.ca/health-care-professionals/clinical-practice-guidelines/exercise-recommendations/. Published 2014.

[12] Parkinson Society Canada. Physical Activity and Parkinson’s Disease. 2012. https://www.csep.ca/CMFiles/Guidelines/specialpops/PSC_Physical_Activity_resource_and_chart_final English march2012.pdf.

[13] Best KL, Arbour-Nicitopoulos KP, Sweet SN (2017). Community-based physical activity and wheelchair mobility programs for individuals with spinal cord injury in Canada: current reflections and future directions. J Spinal Cord Med.

[14] Public Health Agency of Canada. At-a-glance - How healthy are Canadians? A brief update. https://www.canada.ca/en/public-health/services/reports-publications/health-promotion-chronic-disease-prevention-canada-research-policy-practice/vol-38-no-10-2018/at-a-glance-healthy-canadians-update.html. Published 2019.

[15] Casey B, Coote S, Galvin R, Donnelly A (2018). Objective physical activity levels in people with multiple sclerosis: meta-analysis. Scand J Med Sci Sport.

[16] Gaston A, Vamos CA (2013). Leisure-time physical activity patterns and correlates among pregnant women in Ontario Canada. Matern Child Health J.

[17] Goodwin VA, Richards SH, Taylor RS, Taylor AH, Campbell JL (2008). The effectiveness of exercise interventions for people with Parkinson’s disease: a systematic review and meta-analysis. Mov Disord.

[18] Martin Ginis KA, Latimer AE, Arbour-Nicitopoulos KP (2010). Leisure time physical activity in a population-based sample of people With spinal cord injury Part I: demographic and injury-related correlates. Arch Phys Med Rehabil.

[19] Neil SE, Gotay CC, Campbell KL (2014). Physical activity levels of cancer survivors in Canada: findings from the Canadian community health survey. J Cancer Surviv.

[20] Tudor-Locke C, Craig CL, Aoyagi Y (2011). How many steps/day are enough? For older adults and special populations. Int J Behav Nutr Phys Act.

[21] Warburton DER, Bredin SSD (2017). Health benefits of physical activity: a systematic review of current systematic reviews. Curr Opin Cardiol.

[22] Rabin BA, Brownson RC, Haire-Joshu D, Kreuter MW, Weaver NL (2008). A glossary for dissemination and implementation research in health. J Public Heal Manag Pract.

[23] Tomasone JR, Kauffeldt KD, Morgan TL (2020). Dissemination and implementation of national physical activity, sedentary behaviour, and/or sleep guidelines among community-dwelling adults aged 18 years and older: a systematic scoping review and suggestions for future reporting and research. Appl Physiol Nutr Metab.

[24] Morris S, Fawcett G, Brisebois L, Hughes J. A Demographic, Employment and Income Profile of Canadians with Disabilities Aged 15 Years and Over.; 2018. https://www150.statcan.gc.ca/n1/pub/89-654-x/89-654-x2018002-eng.htm.

[25] Houlden RL (2018). 2018 Clinical practice guidelines: introduction. Can J Diabetes.

[26] Williamson C, Baker G, Mutrie N, Niven A, Kelly P. Get the message? A scoping review of physical activity messaging. Int J Behav Nutr Phys Act. 2020;17:1-15. 10.1186/s12966-020-00954-3 PMC7160981 32295613

[27] Latimer-Cheung AE, Martin Ginis KA, Hicks AL (2013). Development of evidence-informed physical activity guidelines for adults with multiple sclerosis. Arch Phys Med Rehabil.

[28] Martin Ginis KA, Van Der Scheer JW, Latimer-Cheung AE (2018). Evidence-based scientific exercise guidelines for adults with spinal cord injury: an update and a new guideline. Spinal Cord.

[29] Bull FC, Al-Ansari SS, Biddle S (2020). World Health Organization 2020 guidelines on physical activity and sedentary behaviour. Br J Sports Med.

[30] D’Urzo KA, Man KE, Bassett-Gunter RE, Latimer-Cheung AE, Tomasone JR (2018). Identifying “real-world” initiatives for knowledge translation tools: a case study of community-based physical activity programs for persons with physical disability in Canada. Transl Behav Med.

[31] Peters MDJ, Godfrey CM, Khalil H, McInerney P, Parker D, Soares CB (2015). Guidance for conducting systematic scoping reviews. Int J Evid Based Healthc.

[32] Levac D, Colquhoun H, O’Brien KK (2010). Scoping studies: advancing the methodology. Implement Sci.

[33] Tricco AC, Lillie E, Zarin W (2018). PRISMA extension for scoping reviews (PRISMA-ScR): checklist and explanation. Ann Intern Med.

[34] National Academy of Sciences. Clinical Practice Guidelines We Can Trust - Institute of Medicine. (Graham R, Mancher M, Miller Wolman D, Greenfield S, Steinberg E, eds.). The National Academies Press; 2011. http://www.nap.edu/catalog.php?record_id=13058%0Ahttps://www.awmf.org/fileadmin/user_upload/Leitlinien/International/IOM_CPG_lang_2011.pdf. 24983061

[35] Neiger BL, Thackeray R, van Wagenen SA (2012). Use of social media in health promotion: purposes, key performance indicators, and evaluation metrics. Health Promot Pract.

[36] Harzing AW (2007). Publish or Perish.

[37] Veritas Health Innovation. Covidence systematic review software. www.covidence.org.

[38] Leeman J, Birken SA, Powell BJ, Rohweder C, Shea CM (2017). Beyond “implementation strategies”: classifying the full range of strategies used in implementation science and practice. Implement Sci.

[39] Corey E (2011). Formative research: what why and how.

[40] Antflick J (2014). End-User Evaluation of Boost Your Brain and Body Power Toolkit [Word Document].

[41] Antflick J. Physician Evaluation of PA-AD Guide [Word Document].

[42] Latimer-Cheung AE, Arbour-Nicitopoulos KP, Brawley LR (2013). Developing physical activity interventions for adults with spinal cord injury. Part 2: motivational counseling and peer-mediated interventions for people intending to be active. Rehabil Psychol.

[43] Shirazipour CH (2013). MS Get Fit Toolkit.

[44] Latimer-Cheung AE, Martin Ginis K. E-Learning Module: MS Physical Activity Guidelines Objectives [PDF Document]. n.d.

[45] Latimer-Cheung AE (2013). MS Get Fit Toolkit consensus meeting [Powerpoint presentation].

[46] Mazza D, Bairstow P, Buchan H (2013). Refining a taxonomy for guideline implementation: results of an exercise in abstract classification. Implement Sci.

[47] Kroshus E, Buth D, Parsons JT, Hainline B (2019). Randomized evaluation of the National Collegiate Athletic Association’s concussion education fact sheet for coaches. Heal Educ Behav.

[48] Canadian Society for Exercise Physiology. Scientific Statement: Canadian Physical Activity Guidelines for Adults with Multiple Sclerosis. http://www.csep.ca/CMFiles/Guidelines/specialpops/CSEP_MS_PAGuidelines_Scientific_Statements_en.pdf. Accessed 27 Nov 2020.

[49] Diabetes Canada. Benefits of physical activity. 10.2466/pms.1997.84.3.890 10.2466/pms.1997.84.3.890 9172199

[50] Campbell K, Winters-Stone KM. An executive summary of reports from an international multidisciplinary roundtable on exercise and cancer: evidence guidelines and implementation. https://journals.lww.com/rehabonc/pages/podcastepisodes.aspx?podcastid=1. Published 2019. Accessed 28 Nov 2020. 10.1097/01.reo.0000000000000186 PMC13606015 42787822

[51] Campbell K. @KLCampbellPhD. https://twitter.com/KLCampbellPhD/status/1186059643048493057. Published 2019. Accessed 30 Nov 2020.

[52] Clark RE, McArthur C, Papaioannou A (2017). “I do not have time. Is there a handout I can use?”: combining physicians’ needs and behavior change theory to put physical activity evidence into practice. Osteoporos Int.

[53] SCI Action Canada. Physical activity guidelines for adults with spinal cord injury: Key messages. https://www.csep.ca/CMFiles/Guidelines/specialpops/SCIPAGuidelinesKey messages_FINAL.pdf. Published 2011. Accessed 27 Nov 2020.

[54] SCI Action Canada. SCI get fit toolkit. https://pace.mcmaster.ca/uploads/Brochures/SCI-Get-Fit-Toolkit-brochure.pdf. Published 2011. Accessed 27 Nov 2020.

[55] Diabetes Canada. Resistance exercise videos. https://www.diabetes.ca/managing-my-diabetes/tools---resources/resistance-exercise-videos. Accessed 28 Nov 2020.

[56] American College of Sports Medicine. Effects of exercise on health-related outcomes in those with cancer. https://www.acsm.org/docs/default-source/files-for-resource-library/exercise-guidelines-cancer-infographic.pdf?sfvrsn=c48d8d86_4. Published 2019. Accessed 28 Nov 2020.

[57] American College of Sports Medicine (2018). Moving through cancer: Exercise for people living with and beyond cancer.

[58] Sunnybrook Odette Cancer Centre. Patient information handout: Exercise for people with cancer. https://www.cancercareontario.ca/sites/ccocancercare/files/assets/H-PSOSunnybrookExerciseForPeopleWithCancerPatientInformationHandout.pdf. Published 2016. Accessed 30 Nov 2020.

[59] Trillium Health Partners. Exercise considerations specific to cancer type. https://www.cancercareontario.ca/sites/ccocancercare/files/assets/H-PSOProgramExerciseConsiderationsSpecificToCancerType2.pdf. Accessed 30 Nov 2020.

[60] Cancer Care Ontario. Exercise for people with cancer. https://www.cancercareontario.ca/sites/ccocancercare/files/assets/H-PSOEExerciseforPeople.pdf. Accessed 30 Nov 2020.

[61] Cancer Care Ontario. Did you know? https://www.cancercareontario.ca/sites/ccocancercare/files/assets/H-PSOExcercisePosterOfficePRINT.pdf. Accessed 30 Nov 2020.

[62] Cancer Care Ontario. Exercise for people with cancer. https://www.cancercareontario.ca/sites/ccocancercare/files/assets/H-PSOEExerciseforPeople.pdf.

[63] Ontario Brain Institute. Boost your brain and body power: Physical activity and Alzheimer’s disease. https://braininstitute.ca/docs/pa-ad_information_pamphlet_english_170310_115017.pdf. Published 2014. Accessed 28 Nov 2020.

[64] Grimes D, Fitzpatrick M, Gordon J, et al. Canadian guideline for Parkinson disease. Can Med Assoc J. 2019;191:E89-1004. 10.1503/cmaj. 10.1503/cmaj.181504 PMC6733687 31501181

[65] Maclaren K. Physical activity throughout pregnancy enhances physical and mental health and reduces risk of pregnancy complications. https://csep.ca/news.asp?a=view&id=202&pageToView=1. Published 2018. Accessed 27 Nov 2020.

[66] SCI Action Canada. Physical activity guidelines for adults with spinal cord injury. https://sciguidelines.ubc.ca/files/2019/06/1_SCI-Physical-Activity-Guidelines.pdf. Published 2019. Accessed 28 Nov 2020.

[67] SCI Action Canada. Physical activity guidelines for adults with spinal cord injury: FAQs. https://sciguidelines.ubc.ca/files/2019/08/2_SCI-Physical-Activity-Guidelines-EN-2019.pdf. Published 2019. Accessed 28 Nov 2020.

[68] Canadian Society for Exercise Physiology. Canadian Physical Activity Guidelines for Adults with Multiple Sclerosis - Questions and Answers. www.csep.ca/guidelines. Accessed 27 Nov 2020.

[69] Multiple Sclerosis Society of Canada. MS Get Fit Toolkit. https://mssociety.ca/en/pdf/MS-Get-Fit-Toolkit.pdf.

[70] Parkinson Canada. From Sandie’s desk: Thou shalt exercise and thou shalt feel better – use it or lose it! https://www.parkinson.ca/wp-content/uploads/Thou-Shalt-Exercise.pdf. Published 2015. Accessed 28 Nov 2020.

[71] Parkinson Canada. Parkinson’s disease: An introductory guide. https://www.parkinson.ca/about-parkinsons/parkinsons-disease-an-introductory-guide/?highlight=%5Bmessag%2A OR guide%2A OR recommend%2A%5D AND %5Bphysical activity OR exercise%5D AND dissem%2A. Published 2018. Accessed 28 Nov 2020.

[72] Parkinson Canada. Care partnering: Managing Parkinson’s disease together. https://www.parkinson.ca/wp-content/uploads/Care_Partnering_Managing_Parkinsons_Disease_Together.pdf. Accessed 28 Nov 2020.

[73] Exercise is Medicine. Exercise for cancer prevention and treatment. https://www.acsm.org/docs/default-source/files-for-resource-library/exercise-for-cancer-prevention-and-treatment-infographic.pdf?sfvrsn=ad47b1e1_2. Accessed 27 Nov 2020.

[74] SCI Action Canada. Scientific exercise guidelines for adults with spinal cord injury. 2018. http://fhsd-sciactioncanada-2019.sites.olt.ubc.ca/files/2019/12/English_The-Spinal-cord-injury-guidelines-BLUE.pdf. 10.1038/s41393-017-0017-3 29070812

[75] Osteoporosis Canada. Too fit to fracture: Managing osteoporosis through exercise. 10.1016/j.jphys.2017.04.003

[76] Wakefield M, Loken B, Hornik RC (2010). Use of mass media campaigns to change health behaviour. Lancet.

[77] Canadian Society for Exercise Physiology [@CSEPdotCA]. Tweets [Twitter profile]. 2019. https://twitter.com/CSEPdotCA?ref_src=twsrc%5Egoogle%7Ctwcamp%5Eserp%7Ctwgr%5Eauthor.

[78] Canadian Society for Exercise Physiology [@csep_scpe]. Posts [Instagram profile]. 2019. https://www.instagram.com/csep_scpe/.

[79] Cancer Care Ontario (2015). Let’s get moving: Exercise and rehabilitation for cancer patients [event].

[80] Devlin M. UBC researchers develop “exercise prescriptions” for cancer survivors. https://bc.ctvnews.ca/ubc-researchers-develop-exercise-prescriptions-for-cancer-survivors-1.4641376. Published 2019. Accessed 30 Nov 2020.

[81] Hutchinson A. Exercise can help in the fight against cancer, but how do we persuade patients to do it? https://www.theglobeandmail.com/life/health-and-fitness/article-exercise-can-help-in-the-fight-against-cancer-but-how-do-we-persuade/. Published 2020. Accessed 30 Nov 2020.

[82] Reynolds G. Exercise advice for surviving cancer, and maybe avoiding it. https://www.nytimes.com/2019/10/16/well/move/exercise-advice-for-surviving-cancer-and-maybe-avoiding-it.html. Published 2019. Accessed 30 Nov 2020.

[83] Segal R. Exercise & enhancing health outcomes in cancer survivors: CCO exercise guidelines. https://www.cancercareontario.ca/sites/ccocancercare/files/assets/H-PSOEDrSegalsPresentationfromPCCRounds.pdf. Published 2017. Accessed 30 Nov 2020.

[84] Grimshaw JM, Thomas RE, MacLennan G, et al. Effectiveness and efficiency of guideline dissemination and implementation strategies. Health Technol Assess. 2004;8(6). 10.1080/04345546709415241 10.3310/hta8060 14960256

[85] Diabetes Canada. Introductory resistance program. https://www.diabetes.ca/diabetescanadawebsite/media/managing-my-diabetes/tools and resources/introductory-resistance-program.pdf?ext=.pdf. Accessed 27 Nov 2020.

[86] Diabetes Canada. Maintaining aerobic exercise. https://www.diabetes.ca/diabetescanadawebsite/media/managing-my-diabetes/tools and resources/maintaining-aerobic-exercise.pdf?ext=.pdf. Accessed 27 Nov 2020.

[87] Diabetes Canada. Planning for regular physical activity. 10.2337/diaclin.23.4.160

[88] Diabetes Canada. Resistance exercise. 10.3109/9780203089798-40

[89] Lithopoulos A, Bassett-Gunter RE, Martin Ginis KA, Latimer-Cheung AE (2017). The effects of gain- versus loss-framed messages following health risk information on physical activity in individuals with multiple sclerosis. J Health Commun.

[90] Shirazipour CH, Tomasone JR, Martin Ginis KA (2019). Enhancing health care professionals’ and trainees’ knowledge of physical activity guidelines for adults with and without SCI. J Spinal Cord Med.

[91] Smith B, Tomasone JR, Latimer-Cheung AE, Martin Ginis KA (2015). Narrative as a knowledge translation tool for facilitating impact: translating physical activity knowledge to disabled people and health professionals. Heal Psychol.

[92] Ma JK, Martin Ginis KA, Cheifetz O. The ProACTIVE SCI toolkit: A physiotherapist’s guide to promoting physical activity to clients who have spinal cord injuries. http://fhsd-sciactioncanada-2019.sites.olt.ubc.ca/files/2019/12/ProacTive_SCI_Toolkit_Nov.pdf. Published 2018. Accessed 27 Nov 2020.

[93] SCI Action Canada. Active homes: Home strength-training guide for people with paraplegia. https://www.themiamiproject.org/wp-content/uploads/2015/07/home-strength-training-guide-paraplegia.pdf. Accessed 27 Nov 2020.

[94] Osteoporosis Canada. Too fit to fall or fracture. http://www.osteoporosis.ca/wp-content/uploads/OC-Too-Fit-to-Fall-or-Fracture.pdf?_ga=2.132151566.1251493687.1574652483-1002885934.1569270259. Accessed 27 Nov 2020.

[95] Community for Advancing Discovery Research in Education. Dissemination toolkit. http://cadrek12.org/dissemination-toolkit. Published 2020.

[96] Boaz A, Hanney S, Borst R, O’Shea A, Kok M (2018). How to engage stakeholders in research: design principles to support improvement. Heal Res Policy Syst.

[97] Gainforth HL, Latimer-Cheung AE, Davis C, Casemore S, Martin Ginis KA (2015). Testing the feasibility of training peers with a spinal cord injury to learn and implement brief action planning to promote physical activity to people with spinal cord injury. J Spinal Cord Med.

[98] Ma JK, West CR, Martin Ginis KA (2019). The effects of a patient and provider co-developed, behavioral physical activity intervention on physical activity, psychosocial predictors, and fitness in individuals with spinal cord injury: a randomized controlled trial. Sport Med.

[99] Latimer-Cheung AE (2013). Agenda and Objectives: MS Get Fit Toolkit Consensus Meeting [Word Document].

[100] Parkinson Canada. Sault Ste. Marie – Dancing with Parkinson’s. https://parkinsonca.thedev.ca/event/sault-ste-marie-dancing-with-parkinsons/. Accessed 28 Nov 2020.

[101] Santa Mina D, Au D, Auger LE (2019). Development, implementation, and effects of a cancer center’s exercise-oncology program. Cancer.

[102] Santa Mina D, Au D, Brunet J (2017). Effects of the community-based wellspring cancer exercise program on functional and psychosocial outcomes in cancer survivors. Curr Oncol.

[103] Trinh L, Arbour-Nicitopoulos KP, Sabiston CM (2018). RiseTx: Testing the feasibility of a web application for reducing sedentary behavior among prostate cancer survivors receiving androgen deprivation therapy. Int J Behav Nutr Phys Act.

[104] Michie S, Johnston M, Francis J, Hardeman W, Eccles M (2008). From theory to intervention: mapping theoretically derived behavioural determinants to behaviour change techniques. Appl Psychol.

[105] Wattanapisit A, Tuangratananon T, Thanamee S (2018). Physical activity counseling in primary care and family medicine residency training: a systematic review. BMC Med Educ.

[106] Arbour-Nicitopoulos KP, Tomasone JR, Latimer-Cheung AE, Martin Ginis KA (2014). Get in motion: an evaluation of the reach and effectiveness of a physical activity telephone counseling service for Canadians living with spinal cord injury. Phys Med Rehabil.

[107] Salci LE, Perrier MJ, Ginis S, Martin Ginis KA (2016). Active living leaders training program for adults with spinal cord injury: a pilot study. Spinal Cord.

[108] Tomasone JR, Arbour-Nicitopoulos KP, Latimer-Cheung AE, Martin Ginis KA (2018). The relationship between the implementation and effectiveness of a nationwide physical activity telephone counseling service for adults with spinal cord injury. Disabil Rehabil.

[109] Chemtob K, Rocchi M, Arbour-Nicitopoulos KP, Kairy D, Fillion B, Sweet SN (2019). Using tele-health to enhance motivation, leisure time physical activity, and quality of life in adults with spinal cord injury: a self-determination theory-based pilot randomized control trial. Psychol Sport Exerc.

[110] Vallerand JR, Rhodes RE, Walker GJ, Courneya KS (2019). Social cognitive effects and mediators of a pilot telephone counseling intervention to increase aerobic exercise in hematologic cancer survivors. J Phys Act Heal.

[111] McMaster University. MS toolkit e-learning module. https://pace.mcmaster.ca/mstoolkit/story_html5.html.

[112] Multiple Sclerosis Society of Canada. Physical activity. https://mssociety.ca/support-services/programs-and-services/recreation-and-social-programs/physical-activity.

[113] Multiple Sclerosis Society of Canada. MS Get Fit Toolkit: Planning sheets. https://mssociety.ca/library/document/1ITihMBaEktnAJRfKxzHqp04OFcr2CLs/original.pdf.

[114] Cancer Care Ontario. Setting exercise goals. https://www.cancercareontario.ca/sites/ccocancercare/files/assets/ExerciseGuide2.pdf.

[115] Ontario Brain Institute. Challenge yourself to move. https://braininstitute.ca/docs/pa_and_ad_calendar_english.pdf. Published 2014. Accessed 28 Nov 2020.

[116] Ondieki WM (2016). Stakeholders’ capacity building and participation in monitoring and evaluation of urban water supply and health projects in Kenya: case of Kisii Town Kisii Country. J Geogr Nat Disasters.

[117] Tomasone JR, Martin Ginis KA, Estabrooks PA, Domenicucci L (2014). “Changing Minds”: Determining the effectiveness and key ingredients of an educational intervention to enhance healthcare professionals’ intentions to prescribe physical activity to patients with physical disabilities. Implement Sci.

[118] Tomasone JR, Martin Ginis KA, Estabrooks PA, Domenicucci L (2015). Changing minds, changing lives from the top down: an investigation of the dissemination and adoption of a Canada-wide educational intervention to enhance health care professionals’ intentions to prescribe physical activity. Int J Behav Med.

[119] Tomasone JR, Sweet SN, McReynolds S, Martin Ginis KA (2017). A multilevel modeling approach to examining the implementation-effectiveness relationship of a behavior change intervention for health care professional trainees. Transl Behav Med.

[120] Schmitz KH, Campbell AM, Stuiver MM (2019). Exercise is medicine in oncology: engaging clinicians to help patients move through cancer. CA Cancer J Clin.

[121] California Social Work Education Center. Implementation toolkits. https://calswec.berkeley.edu/toolkits/implementation-toolkits. Published 2020. Accessed 13 Feb 2020.

[122] Canadian Society for Exercise Physiology. PARmed-X for Pregnancy: Physical activity readiness medical examination. http://www.csep.ca/cmfiles/publications/parq/parmed-xpreg.pdf. Published 2015. Accessed 27 Nov 2020.

[123] Oncology Nursing Society. Physical activity recommendation. https://www.cancercareontario.ca/sites/ccocancercare/files/assets/H-PSOEONSPhysicalActivityRecommendationForm.pdf. Accessed 30 Nov 2020.

[124] Exercise is Medicine. Exercise program registry: Cancer registry program. https://www.exerciseismedicine.org/cancer_exercise.php. Accessed 27 Nov 2020.

[125] Exercise is Medicine. Moving through cancer. https://www.exerciseismedicine.org/assets/page_documents/EIM moving through cancer form web.pdf. Accessed 27 Nov 2020.

[126] Diabetes Canada. Physical activity interactive decision tool. https://www.diabetes.ca/managing-my-diabetes/tools---resources/physical-activity-interactive-decision-tool. Accessed 27 Nov 2020.

[127] SCI Action Canada. Active living leaders. http://sciactioncanada.ca/active-living-leaders. Accessed 27 Nov 2020.

[128] Higgins JPT, Altman DG, Gøtzsche PC (2011). The cochrane collaboration’s tool for assessing risk of bias in randomised trials. BMJ.

[129] Sterne J, Higgins J, Reeves B, on behalf of the development group for ACROBAT-NRSI. A Cochrane Risk of Bias Assessment Tool: for Non-Randomized Studies of Interventions (ACROBAT-NRSI). Version 1.0.0. 2014;September. http://www.riskofbias.info.

[130] Latimer-Cheung AE. MS Get Fit Toolkit Stakeholder Feedback Comments - English [Word Document]. n.d.

[131] Center for Community Health and Development. Section 15. Creating fact sheets on local issues. https://ctb.ku.edu/en/table-of-contents/participation/promoting-interest/fact-sheets/main. Published 2020.

[132] Payne J, France K, Henley N (2011). Changes in health professionals’ knowledge, attitudes and practice following provision of educational resources about prevention of prenatal alcohol exposure and fetal alcohol spectrum disorder. Paediatr Perinat Epidemiol.

[133] Price SM, Bonilla E, Zador P, Levis DM, Kilgo CL, Cannon MJ (2014). Educating women about congenital cytomegalovirus: assessment of health education materials through a web-based survey. BMC Womens Health.

[134] Bland KA, Bigaran A, Campbell KL, Trevaskis M, Zopf EM (2020). Exercising in isolation? The role of telehealth in exercise oncology during the COVID-19 pandemic and beyond. Phys Ther.

[135] Innes AQ, Thomson G, Cotter M, King JA, Vollaard NBJ, Kelly BM (2019). Evaluating differences in the clinical impact of a free online weight loss programme. BMC Public Health.

[136] Hagger MS, Luszczynska A (2014). Implementation intention and action planning interventions in health contexts: state of the research and proposals for the way forward. Appl Psychol Heal Well-Being.

[137] Elouafkaoui P, Young L, Newlands R (2016). An audit and feedback intervention for reducing antibiotic prescribing in general dental practice: the RAPiD cluster randomised controlled trial. PLoS Med.

[138] Gagliardi AR, Brouwers MC, Bhattacharyya OK (2014). A framework of the desirable features of guideline implementation tools (GItools): Delphi survey and assessment of GItools. Implement Sci.

[139] Rogers EM (2003). Diffusion of innovations.

[140] Kramer DM, Wells RP, Bigelow PL, Carlan NA, Cole DC, Hepburn CG (2010). Dancing the two-step: collaborating with intermediary organizations as research partners to help implement workplace health and safety interventions. Work.

[141] Broemeling A, Watson DE, Prebtani F, Outcomes H (2008). Population patterns of chronic health conditions, co-morbidity and healthcare use in Canada: Implications for policy and practice. Healthc Q.

[142] Tomasone JR, Flood SM, Latimer-Cheung AE (2020). Knowledge translation of the Canadian 24-hour movement guidelines for adults aged 18–64 years and adults aged 65 years or older: a collaborative movement guideline knowledge translation process. Appl Physiol Nutr Metab.

[143] Graham ID, Kothari A, McCutcheon C (2018). Moving knowledge into action for more effective practice, programmes and policy: protocol for a research programme on integrated knowledge translation. Implement Sci.

[144] Wilroy JD, Lai B, Davlyatov G, Mehta T, Thirumalai M, Rimmer JH (2020). Correlates of adherence in a home-based, self-managed exercise program tailored to wheelchair users with spinal cord injury. Spinal Cord.

[145] Martin Ginis KA, Hicks AL (2005). Exercise research issues in the spinal cord injured population. Exerc Sport Sci Rev.

