# Supplementary material for: Dissemination and implementation strategies for physical activity guidelines among adults with disability, chronic conditions, and pregnancy: a systematic scoping review
Source: BMC Public Health. 2022 May 24;22:1034. doi: 10.1186/s12889-022-13317-3 (PMC9126633; doi:10.1186/s12889-022-13317-3)
Supplement: Supplementary file 1 — Additional file 1:Supplement 1. Search terms for guideline identification, database searches, and targeted web-based search strings. Supplement 2. Risk of bias assessment for included randomized controlled trials. Supplement 3. Risk of bias assessment for included non-randomized interventions. [file 12889_2022_13317_MOESM1_ESM.docx]

**Supplemental Material and Figures**

**Supplement 1.** Search terms for guideline identification, database searches, and targeted web-based search strings.

|  | **Guideline identification** | **Database searches^a^** | **Targeted web-based search strings** |
| --- | --- | --- | --- |
| Search strategy | “physical activity guidelines OR recommendations for ___________ Canada” for cancer, MS, pregnancy, Parkinson’s disease, osteoporosis, osteoarthritis, diabetes, Alzheimer’s, and SCI. | (Change* or Information or Policy or Evaluation or Communication or Policies or Implementation or Utili?ation or Adoption or Validation or "Organi?ational innovation" or Spread or Innovation or “Quality improvement” or Dissemination or Diffusion or “Best practice*” or Transfer or “Patient safety” or Translation or “Continuing education” or “"Complex intervention*"”or “Total quality management” or Sustainability or "Implementation n/3 research" or "Research utili?ation" or Institutionali?ation or “Diffusion of innovation*” or “Action research” or “Translational research” or “Quality assurance” or “Participatory research” or “Knowledge management” or “Communit* of Practice” or “Capacity building” or “Technology transfer” or “Sociology of Knowledge” or “Service Innovation” or “Research capacity” or “Policy Research” or “Opinion leader*” or “Knowledge transfer” or “Knowledge diffusion” or "Knowledge n/3 (utili?ation or synthesis or dissemination)" or “Information Science” or "Effectiveness n/3 research")  ^a^ These search terms were combined with terms for physical activity (i.e., “physical activity OR exercise OR movement OR exercise therapy OR fitness”) and terms for the specific populations (i.e., “cancer”, “multiple sclerosis OR MS”, “pregnancy”, “Parkinson’s disease”, “osteoporosis”, “osteoarthritis”, “diabetes”, “Alzheimer’s”, and “spinal cord injury OR SCI”). | “[messag* OR guide* OR recommend*] AND [physical activity OR exercise] AND dissem*” and “[messag* OR guide* OR recommend*] AND [physical activity OR exercise] AND implem*” were the search strings run through each website. |

**Supplement 2.** Risk of bias assessment for included randomized controlled trials.

| **Author** | **Overall risk of bias** | **Random sequence generation (selection bias)** | **Allocation concealment (selection bias)** | **Blinding of participants and personnel (performance bias)** | **Blinding of outcome assessment (detection bias)** | **Incomplete outcome data (attrition bias)** | **Selective reporting (reporting bias)** | **Other bias** |
| --- | --- | --- | --- | --- | --- | --- | --- | --- |
| Chemtob et al, 2019 | **High** | Low | Low | High | High | High | Low | Low |
| Lithopoulos et al., 2017 | **High** | Unclear | Unclear | High | High | High | Low | High |
| Ma et al., 2019 | **High** | Low | High | High | High | High | Low | Low |
| Vallerand et al., 2019 | **High** | High | Unclear | High | High | Low | Low | Unclear |

**Supplement 3.** Risk of bias assessment for included non-randomized interventions.

| **Author** | **Overall Risk of Bias** | **Bias due to confounding** | **Bias in selection of participants** | **Bias in measurement of interventions** | **Bias due to departures from intended interventions** | **Bias due to missing data** | **Bias in measurement of outcomes** | **Bias in selection of reported results** |
| --- | --- | --- | --- | --- | --- | --- | --- | --- |
| Trinh et al., 2018 | **Serious** | Serious | Serious | Moderate | Unclear | Moderate | Moderate | Low |
| Santa Mina et al., 2019 | **Serious** | Serious | Serious | Moderate | Unclear | Moderate | Serious | Low |
| Salci et al., 2016 | **Serious** | Serious | Serious | Unclear | Unclear | Unclear | Unclear | Low |
| Santa Mina et al., 2017 | **Serious** | Serious | Low | Serious | Unclear | Serious | Serious | Low |
| Gainforth et al., 2015 | **Serious** | Serious | Serious | Unclear | Unclear | Low | Serious | Low |
| Tomasone et al., 2015 | **Serious** | Serious | Unclear | Low | Unclear | Unclear | Unclear | Low |
| Tomasone et al., 2014 | **Serious** | Serious | Low | Low | Unclear | Unclear | Serious | Low |
| Arbour-Nicitopoulos et al., 2014 | **Serious** | Serious | Low | Serious | Unclear | Low | Serious | Low |
| Latimer-Cheung et al., 2013 | **Serious** | Serious | Low | Serious | Unclear | Low | Serious | Low |
| Smith et al., 2014 | **Serious** | Serious | Low | Low | Unclear | Unclear | Serious | Unclear |
| Shirazipour et al, 2019 | **Serious** | Serious | Serious | Low | Unclear | Serious | Serious | Low |
| Tomasone et al., 2018 | **Serious** | Serious | Low | Serious | Unclear | Serious | Unclear | Low |
| Tomasone et al., 2017 | **Serious** | Serious | Unclear | Low | Unclear | Unclear | Unclear | Low |
| Antflick, 2014 | **Serious** | Unclear | Unclear | Unclear | Unclear | Serious | Unclear | Unclear |
| Antflick, n.d. | **Serious** | Unclear | Unclear | Unclear | Unclear | Serious | Unclear | Unclear |
| Latimer-Cheung, n.d. | **Serious** | Unclear | Unclear | Unclear | Unclear | Serious | Unclear | Unclear |

**Figure 1.** PRISMA diagram of study flow*^a^*
